# Supplementary material for: Characterisation of populations at risk of sub-optimal dosing of artemisinin-based combination therapy in Africa
Source: PLOS Glob Public Health. 2023 Dec 1;3(12):e0002059. doi: 10.1371/journal.pgph.0002059 (PMC10691722; doi:10.1371/journal.pgph.0002059)
Supplement: S1 Text — (DOCX) [file pgph.0002059.s001.docx]

**Characterisation of populations at risk of sub-optimal dosing of artemisinin-based combination therapy in Africa**

**Methods** **for** **estimation of number/proportion of malaria cases in risk groups**

[**1.** **Country selection – exclusion criteria** 2](#_Toc150277464)

[**Table A. Countries excluded from analysis** 2](#_Toc150277465)

[**2.** **Data sources** 2](#_Toc150277466)

[**Table B. Country-level variables selected and their sources** 3](#_Toc150277467)

[**3.** **Calculations** 5](#_Toc150277468)

[**A.** **Number of pregnant women per country** 5](#_Toc150277469)

[**B.** **Risk Ratio urban/rural for 4 sub-populations at risk of malaria** 5](#_Toc150277470)

[**Table C. Risk Ratio urban/rural for 4 sub-populations at risk of malaria** 6](#_Toc150277471)

[**C. Calculation of the number of people with malaria and characteristic γ in country X** 7](#_Toc150277472)

[**4.** **References** 9](#_Toc150277473)

# **Country selection – exclusion criteria**

## **Table A. Countries excluded from analysis**

| **Reason** | **Countries** |
| --- | --- |
| No indigenous malaria transmission | Morocco ; Tunisia; Libya; Egypt; Lesotho; Mauritius; Seychelles ; La Réunion ; Mayotte ; St Helena |
| Countries participating to the E2020 initiative [1] | Algeria; Botswana; Cabo Verde; Comoros; South Africa; Eswatini |

# **Data sources**

Country-level data were extracted from seven public, openly accessible data sources: Malaria Atlas Project [2], United Nations Department of Economic and Social Affairs/Population Division (UNDP) [3], United Nations Department of Economic and Social Affairs/Statistics Division [4], The World Bank [5], Global Health Observatory (GHO) [6], WHO World Malaria Report 2021 [7], and UNAIDS [8]. Twenty-seven variables were retrieved which included malaria indicators, population by gender, age-group, nutritional status and rural or urban areas, as well as the number of People Living with HIV (PLHIV) and fertility data (Table B).

Number of confirmed uncomplicated malaria cases was calculated as the number of confirmed malaria cases by microscopy and/or rapid diagnostic tests from the WHO World Malaria Report 2021 minus the number of severe malaria cases derived from the work from Camponovo *et al.* who provided estimates of inpatient severe malaria cases per 100000 persons for 41 African countries, 39 of which are part of this analysis [9]. Severe malaria cases for countries without available data were estimated using the median proportion of severe cases in countries from the same region (defined as per United Nations M49 Standards) [4].

The proportion of patients with hyperparasitaemia was assumed to be the same for each population category and for rural or urban setting, and based on the proportion of 10.2% derived from an individual patient meta-analysis of over 50000 patients from 29 African countries [10].

## **Table B. Country-level variables selected and their sources**

| **Type of Data** | **Variable/ field name** | **Variable description** | **Unit** | **Sources** | **Year available** | **Reference link** |
| --- | --- | --- | --- | --- | --- | --- |
|  | Region | Standard country or area codes for statistical use (M49): African regions | .. | UN statistics division |  | <https://unstats.un.org/unsd/methodology/m49/> |
| Malaria data | prev | age-standardized parasite rate for *Plasmodium falciparum* malaria for children two to ten years of age (PfPR2-10) | rate | Malaria Atlas Project | 2020 | <https://malariaatlas.org/> |
| Malaria data | Confirmed RDT cases | Annex 3 – H. Reported malaria cases by method of confirmation, 2010–2020 - RDT positive | Number | World Malaria Report 2021 (Annex 3-H) | 2020 or latest year available | https://www.who.int/teams/global-malaria-programme/reports/world-malaria-report-2021 |
| Malaria data | Confirmed Micro cases | Annex 3 – H. Reported malaria cases by method of confirmation, 2010–2020 - Microscopy positive | Number | World Malaria Report 2021 (Annex 3-H) | 2020 or latest year available | https://www.who.int/teams/global-malaria-programme/reports/world-malaria-report-2021 |
| Malaria data | Inpatient severe malaria incidence (Severe) | Country-specific in-patient severe cases incidence (per 100000 person per year) | per 100000 person-year | Camponovo *et al*. 2017 (Table 2) | 2015 | <https://www.ncbi.nlm.nih.gov/pubmed/28049519> |
| Malaria data | Hyperparasitaemia (Hyper) | Proportion of patients with hyperparasitaemia assumed to be 10.2% and the same for each sub-population group | rate | WWARN Haematology Study Group 2022 | 1991-2013 | https://bmcmedicine.biomedcentral.com/articles/10.1186/s12916-022-02265-9 |
| Malaria data | prop_age | Estimated proportion of malaria cases in each age group (0-5; 5-10; 10-15; 15+) according to prevalence levels | rate | Griffin *et al*. 2013 (Fig 3c) | 2013 | https://www.ncbi.nlm.nih.gov/pubmed/24518518 |
| Population data | Population | Total Population (both sexes combined). De facto population in a country, area or region as of 1 July of the year indicated. Data are presented in thousands. | x1000 | UNPD,World Population Prospects 2022 | 2020 | <https://esa.un.org/unpd/wpp/Download/Standard/Population/> |
| Population data | Rural population (Rural pop) | Rural population (% of the total population): World Bank staff estimates based on the United Nations Population Division's World Urbanization Prospects: 2018 Revision. | % | The World Bank data | 2020 | <https://data.worldbank.org/indicator/SP.RUR.TOTL.ZS?locations=ZG> |
| Population data | Child0-14 M (M0_14) | Male population by select age group. De facto population as of 1 July of the year indicated classified by select age groups. Data are presented in thousands. | x1000 | UNPD,World Population Prospects 2022 | 2020 | <https://esa.un.org/unpd/wpp/Download/Standard/Population/> |
| Population data | Child0-14 F (F0_14) | Female population by select age group. De facto population as of 1 July of the year indicated classified by select age groups. Data are presented in thousands. | x1000 | UNPD,World Population Prospects 2022 | 2020 | <https://esa.un.org/unpd/wpp/Download/Standard/Population/> |
| Population data | Adults18+ M (M18plus) | Male population by select age group. De facto population as of 1 July of the year indicated. Data are presented in thousands. | x1000 | UNPD,World Population Prospects 2022 | 2020 | <https://esa.un.org/unpd/wpp/Download/Standard/Population/> |
| Population data | Adults18+ F (F18plus) | Female population by select age group. De facto population as of 1 July of the year indicated. Data are presented in thousands. | x1000 | UNPD,World Population Prospects 2022 | 2020 | <https://esa.un.org/unpd/wpp/Download/Standard/Population/> |
| Population data | Adults15+ M (M15plus) | Male population by select age group. De facto population as of 1 July of the year indicated. Data are presented in thousands. | x1000 | UNPD,World Population Prospects 2022 | 2020 | <https://esa.un.org/unpd/wpp/Download/Standard/Population/> |

**Table B. Cont.-**

| Population data | Adults15+ F (F15plus) | Female population by select age group. De facto population as of 1 July of the year indicated. Data are presented in thousands. | x1000 | UNPD,World Population Prospects 2022 | 2020 | <https://esa.un.org/unpd/wpp/Download/Standard/Population/> |
| --- | --- | --- | --- | --- | --- | --- |
| Population data | Adults15+ all (Adults15plus) | Total population (both sexes combined) by select age group. De facto population as of 1 July of the year indicated. Data are presented in thousands. | x1000 | UNPD,World Population Prospects 2022 | 2020 | <https://esa.un.org/unpd/wpp/Download/Standard/Population/> |
| Population data | Adults15-49 F (F15to49) | Female population by select age group. De facto population as of 1 July of the year indicated. Data are presented in thousands. | x1000 | UNPD,World Population Prospects 2022 | 2020 | <https://esa.un.org/unpd/wpp/Download/Standard/Population/> |
| Fertility data | Crude birth rate (CBR) | Number of births over a given period divided by the person-years lived by the population over that period. It is expressed as average annual number of births per 1000 population. | per 1000 | UNPD,World Population Prospects 2022 | 2020 | <https://esa.un.org/unpd/wpp/Download/Standard/Population/> |
| Fertility data | Total fertility rate (TFR) | The average number of live births a hypothetical cohort of women would have at the end of their reproductive period if they were subject during their whole lives to the fertility rates of a given period and if they were not subject to mortality. It is expressed as live births per woman. | per woman | UNPD,World Population Prospects 2022 | 2020 | <https://esa.un.org/unpd/wpp/Download/Standard/Population/> |
| Fertility data | Abortion rate (AR) | Estimated abortion rates per 1000 women 15–49 years old, by geographic area and time period | per 1000 | Bearak *et al*. 2020 (Appendix Table 4) | 2015-2019 | <https://www.ncbi.nlm.nih.gov/pubmed/32710833> |
| Nutrition data | U5 wasted | Wasting prevalence among children under 5 years of age (% weight-for-height <-2 SD) (country survey results) | % | GHO | 2020 or latest year available | [Indicators (who.int)](https://www.who.int/data/gho/data/indicators) |
| Nutrition data | Overweight Adults18+ M (Overwt AdultM) | Prevalence of overweight among adults’ males 18+ years, BMI>=25 (age-standardized estimate), (%) | % | GHO | 2016 | [Indicators (who.int)](https://www.who.int/data/gho/data/indicators) |
| Nutrition data | Overweight Adults18+ F (Overw tAdultF) | Prevalence of overweight among adults’ females 18+ years, BMI>=25 (age-standardized estimate), (%) | % | GHO | 2016 | [Indicators (who.int)](https://www.who.int/data/gho/data/indicators) |
| HIV data | HIV Child0-14 (HIV 0_14) | People living with HIV - Children (0-14) | Number | UNAIDS | 2020 | <http://aidsinfo.unaids.org/> |
| HIV data | HIV Adults15+ M (HIV15M) | People living with HIV - Male adults (15+) | Number | UNAIDS | 2020 | <http://aidsinfo.unaids.org/> |
| HIV data | HIV Adults15+ F (HIV15F) | People living with HIV - Female adults (15+) | Number | UNAIDS | 2020 | <http://aidsinfo.unaids.org/> |
| HIV data | HAART | Reported number of people receiving antiretroviral therapy | Number | GHO | 2020 | [Indicators (who.int)](https://www.who.int/data/gho/data/indicators) |

# **Calculations**

### **Number of pregnant women per country**

Number of pregnant women per country is not collected routinely and was estimated as the total of induced abortions, spontaneous foetal loss, and live births. Formula used was similar to that in Dellicour *et al.* [11]

LB live births

IA induced abortions

FL spontaneous foetal loss (including miscarriage and stillbirth)

WOCBA_15-49_ women of child bearing age 15-49 years

AR abortion rate (derived from table 4 of the publication by Bearak *et al.*) [12]

CBR crude birth rate

TFR total fertility rate

Considering that the number of pregnancies = LB + IA + FL

1. Calculating the number of live births per woman

LB = CBR/1000*Country population

1. Calculating the number of induced abortions per woman

IA=AR/1000*WOCBA _15-49_

1. Calculating the number of induced abortions per woman

FL=0.10*IA + 0.20*LB

### **Risk Ratio urban/rural for 4 sub-populations at risk of malaria**

Malaria risk was considered four times higher in rural areas than urban settings based on published entomological inoculation rate estimates [13-15].

Differences in prevalence between urban and rural areas have been reported in the literature and we therefore included a risk ratio in the calculations to estimate the number at risk of malaria among each of the four sub-populations at risk of malaria. Country-specific urban/rural risk in children <5 years of age severely wasted from the publication by Fagbamigbe *et al*. in 2020 was extended to moderately wasted children [16]; rural and urban fertility rate was used as a proxi for pregnant women and data extracted from the USAID STAT compiler website [17]; data from a publication by Garcia-Calleja *et al*. were used to extrapolate urban/rural HIV risk ratio [18]; and overweight urban/rural risk ratio for women from the publication by Jiwani *et al* was extrapolated to the global sub-population, assuming a similar diet and lifestyle between gender [19]. The region risk ratio average was calculated for countries without data and presented in italic in Table C.

### **Table C. Risk Ratio urban/rural for 4 sub-populations at risk of malaria**

| **Country** | **Region** | **U5 wasted** | **Pregnant women** | **PLHIV** | **Adults overweight** |
| --- | --- | --- | --- | --- | --- |
| Angola | Central Africa | 0.57 | 0.65 | *1.37* | *1.83* |
| Benin | Western Africa | 1.00 | 0.85 | *1.95* | 1.65 |
| Burkina Faso | Western Africa | 1.02 | 0.66 | 2.77 | 3.20 |
| Burundi | Eastern Africa | 0.30 | 0.72 | 3.73 | 3.21 |
| Cameroon | Central Africa | 0.30 | 0.63 | 1.68 | 1.43 |
| Central African Republic | Central Africa | *0.50* | 0.94 | *1.37* | *1.83* |
| Chad | Central Africa | 1.07 | 0.79 | *1.37* | 2.50 |
| Congo | Central Africa | 0.94 | 0.69 | *1.37* | 1.86 |
| Côte d'Ivoire | Western Africa | 0.67 | 0.59 | *1.95* | 1.83 |
| Democratic Republic of the Congo | Central Africa | 0.47 | 0.74 | *1.37* | 2.24 |
| Djibouti | Eastern Africa | *0.65* | *0.67* | *2.27* | *2.23* |
| Equatorial Guinea | Central Africa | *0.50* | *0.73* | 1.06 | *1.83* |
| Eritrea | Eastern Africa | *0.65* | 0.61 | *2.27* | *2.23* |
| Ethiopia | Eastern Africa | 0.71 | 0.71 | *2.27* | 5.41 |
| Gabon | Central Africa | 0.69 | 0.64 | *1.37* | 1.14 |
| Gambia | Western Africa | 0.96 | 0.66 | *1.95* | 1.53 |
| Ghana | Western Africa | 0.75 | 0.67 | 1.15 | 1.12 |
| Guinea | Western Africa | 0.59 | 0.66 | 2.40 | 2.20 |
| Guinea-Bissau | Western Africa | *0.88* | *0.67* | *1.95* | *1.83* |
| Kenya | Eastern Africa | 0.80 | 0.75 | 1.79 | 1.53 |
| Liberia | Western Africa | 0.91 | 0.62 | *1.95* | 1.33 |
| Madagascar | Eastern Africa | *0.65* | 0.63 | *2.27* | *2.23* |
| Malawi | Eastern Africa | 1.60 | 0.62 | *2.27* | 1.66 |
| Mali | Western Africa | 0.87 | 0.72 | 1.47 | 1.81 |
| Mauritania | Western Africa | *0.88* | 0.64 | *1.95* | *1.83* |
| Mozambique | Eastern Africa | 0.63 | 0.63 | *2.27* | 2.28 |
| Namibia | Southern Africa | 0.62 | 0.65 | *1.23* | 1.58 |
| Niger | Western Africa | 0.97 | 0.69 | 3.23 | 2.33 |
| Nigeria | Western Africa | 0.93 | 0.76 | *1.95* | 1.70 |
| Rwanda | Eastern Africa | 1.00 | 0.79 | 3.32 | 1.76 |
| Sao Tome and Principe | Central Africa | *0.50* | 0.80 | *1.37* | *1.83* |
| Senegal | Western Africa | 0.56 | 0.66 | 1.00 | 1.87 |
| Sierra Leone | Western Africa | 0.95 | 0.61 | 1.62 | 1.64 |
| Somalia | Eastern Africa | *0.65* | *0.67* | *2.27* | *2.23* |
| South Sudan | Eastern Africa | *0.65* | 0.74 | *2.27* | *2.23* |
| Sudan | Northern Africa | *1.05* | 0.74 | *2.27* | *2.23* |
| Togo | Western Africa | 1.20 | 0.63 | *1.95* | 1.59 |
| Uganda | Eastern Africa | 0.67 | 0.69 | 1.65 | 1.60 |
| United Republic of Tanzania | Eastern Africa | 0.57 | 0.61 | 2.06 | 1.59 |
| Zambia | Eastern Africa | 1.00 | 0.59 | 2.14 | 1.85 |
| Zimbabwe | Eastern Africa | 0.75 | 0.64 | 1.21 | 1.39 |

Values in italic are a risk average of the region as country-specific ratio was not available.

### **C. Calculation of the number of people with malaria and characteristic γ in country X**

To ensure that malaria cases were only classified into one risk group (and therefore counted only once), sequential calculations within each age group were performed in the following order: i) children <5 years of age: wasted, living with HIV, hyperparasitaemic; ii) children 5-14 years of age: living with HIV, hyperparasitaemic iii) adults >14 years: pregnant, living with HIV, overweight, hyperparasitaemic.

Note: symbols in bold represent unknown quantities

$N$ total population

$U$ urban population

$R$ rural population

$\boldsymbol{U}_{\boldsymbol{m}}$ urban population with malaria

$\boldsymbol{R}_{\boldsymbol{m}}$ rural population with malaria

$Nm =\boldsymbol{U}_{\boldsymbol{m}} + \boldsymbol{R}_{\boldsymbol{m}}$ total population with malaria

$\boldsymbol{U}_{\boldsymbol{\gamma}}$ urban population with characteristic γ

$\boldsymbol{R}_{\boldsymbol{\gamma}}$ rural population with characteristic γ

$N\gamma=\boldsymbol{U}_{\boldsymbol{\gamma}}\boldsymbol{+}\boldsymbol{R}_{\boldsymbol{\gamma}}$ total population with characteristic γ

Assuming that risk of malaria in people with characteristic γ is the same as in the general population within each setting:

$\boldsymbol{R}_{\boldsymbol{m\gamma}}\boldsymbol{=}\boldsymbol{R}_{\boldsymbol{\gamma}}/R \times\boldsymbol{R}_{\boldsymbol{m}}/R \times R$ rural population with characteristic γ and malaria

$\boldsymbol{U}_{\boldsymbol{\gamma}}\boldsymbol{=}\boldsymbol{U}_{\boldsymbol{\gamma}}/U \times\boldsymbol{U}_{\boldsymbol{m}}/U \times U$ urban population with characteristic γ and malaria

The total number of individuals with characteristic γ and malaria

$\boldsymbol{N}_{\boldsymbol{m\gamma}}=\left( \frac{1}{R}\times\boldsymbol{R}_{\boldsymbol{\gamma}}\times\boldsymbol{R}_{\boldsymbol{m}} \right)+\left( \frac{1}{U}\times\boldsymbol{U}_{\boldsymbol{\gamma}}\times\boldsymbol{U}_{\boldsymbol{m}} \right)$ (1)

**Scenario 1: Prevalence of characteristic γ is the same in urban and rural areas**

$$\boldsymbol{R\gamma}/R = \boldsymbol{U\gamma}/U = N\gamma/N$$

Substituting to (1):

$$\boldsymbol{Nm\gamma}=\frac{\boldsymbol{N\gamma}}{N}\times\boldsymbol{R}_{\boldsymbol{m}} +\frac{\boldsymbol{N\gamma}}{N}\times\boldsymbol{Um} =\frac{N\boldsymbol{\gamma}}{N}\times\left( \boldsymbol{R}_{\boldsymbol{m}}\boldsymbol{+Um} \right)$$

$\boldsymbol{N}_{\boldsymbol{m\gamma}}=\frac{{N_{m}N}_{\gamma}}{N}$ (2)

**Scenario 2: Prevalence of characteristic γ is different in urban and rural areas**

For malaria we have:

$\boldsymbol{Um}/U = \alpha\times\boldsymbol{R}_{\boldsymbol{m}}/R \boldsymbol{Um}+ \boldsymbol{R}_{\boldsymbol{m}}=Nm$

where α is the ratio of malaria prevalence between urban and rural areas (Risk Ratio)

For characteristic γ we have:

$$\boldsymbol{U}_{\boldsymbol{\gamma}}/U = \beta\times\boldsymbol{R}_{\boldsymbol{\gamma}}/R \boldsymbol{U}_{\boldsymbol{\gamma}}+ \boldsymbol{R}_{\boldsymbol{\gamma}}=N\gamma$$

where β is the ratio of γ prevalence between urban and rural areas (Risk Ratio)

Solving the set of linear equations for malaria, we get:

$$\alpha\times\boldsymbol{R}\boldsymbol{m}/R\times U + \boldsymbol{R}\boldsymbol{m}=Nm$$

$$\Rightarrow\boldsymbol{R}\boldsymbol{m} (\alpha\times U/R+ 1) \boldsymbol{=}Nm$$

$$\boldsymbol{R}_{\boldsymbol{m}}=\frac{RN_{m}}{\alpha U+R}$$

**Um**/U = α × **Rm**/R

$$\Rightarrow\boldsymbol{U}_{\boldsymbol{m}}=\frac{\alpha U}{R}\times\boldsymbol{R}_{\boldsymbol{m}}\Rightarrow\boldsymbol{U}_{\boldsymbol{m}}=\frac{\alpha U}{R}\times\frac{N_{m}}{\left( \frac{\alpha U}{R}+1 \right)}\Rightarrow\boldsymbol{U}_{\boldsymbol{m}}=\frac{\alpha U}{R}\times\frac{N_{m}}{\left( \frac{\alpha U+R}{R} \right)}$$

$$\boldsymbol{U}_{\boldsymbol{m}}=\frac{\alpha UN_{m}}{\alpha U+R}$$

Similarly, for characteristic γ we get:

$$R_{\gamma}=\frac{RN_{\gamma}}{\beta U+R}$$

$$\boldsymbol{U}_{\gamma}=\frac{\beta UN_{\gamma}}{\beta U+R}$$

Subbing in equation (1):

$$\boldsymbol{N}_{\boldsymbol{m\gamma}}=\left\{ \frac{1}{R}\times\left( \frac{{RN}_{\gamma}}{\left( \beta U+R \right)} \right)\times\frac{{RN}_{m}}{\left( \alpha U+R \right)} \right\}+\left\{ \left( \frac{1}{U}\times\frac{\beta UN_{\gamma}}{\left( \beta U+R \right)}\times\frac{{\alpha UN}_{m}}{\left( \alpha U+R \right)} \right) \right\}$$

$$\Rightarrow\boldsymbol{N}_{\boldsymbol{m\gamma}}=\frac{{N_{m}N}_{\gamma}R}{\left( \beta U+R \right)\left( \alpha U+R \right)}+\frac{N_{m}N_{\gamma}\alpha\beta U}{\left( \beta U+R \right)\left( \alpha U+R \right)}$$

$$\Rightarrow\boldsymbol{N}_{\boldsymbol{m\gamma}}=\frac{{N_{m}N}_{\gamma}R+N_{m}N_{\gamma}\alpha\beta U}{\left( \beta U+R \right)\left( \alpha U+R \right)}$$

$\boldsymbol{N}_{\boldsymbol{m\gamma}}=\frac{{N_{m}N}_{\gamma}\left( R+\alpha\beta U \right)}{\left( \beta U+R \right)\left( \alpha U+R \right)}$ (3)

Finally, showing that the derived expression (3) reduces to expression (2) in case of homogenous scenario i.e. $\alpha=1 and \beta=1$ :

$$\boldsymbol{N}_{\boldsymbol{m\gamma}}=\frac{{N_{m}N}_{\gamma}\left( R+U \right)}{\left( U+R \right)\left( U+R \right)}=\frac{{N_{m}N}_{\gamma}}{\left( U+R \right)}=\frac{{N_{m}N}_{\gamma}}{N}$$

**Calculation of β (RR) when odd ratios (OR) are given for characteristic γ in country X**

Risk ratio (RR) comparing urban to rural prevalence can be calculated from OR using the following formula from Grant, 2014 [20]:

$$RR = OR / (1 - p + (p OR))$$

where $p$ is the prevalence in rural area.

Using our notation from the previous section we get:

$\beta= OR / (1 - p + (p OR))$

where ${p=R}_{\gamma}/R={N_{\gamma}}/{(\beta U+R})$

Substituting we get:

$\beta= OR / (1 - \frac{N_{\gamma}}{\beta U+R} + (\frac{N_{\gamma}}{\beta U+R} OR))$

$\beta(1 - \frac{N_{\gamma}}{\beta U+R} + (\frac{N_{\gamma}}{\beta U+R} OR))= OR$

$$\beta(\beta U+R -N_{\gamma}+ N_{\gamma}\times OR)= OR\times\beta U+OR\times R$$

$$\beta^{2}\times U+\beta\left( R -N_{\gamma}+ N_{\gamma}\times OR-OR\times U \right)-OR\times R=0$$

Solving quadratic equation, we get:

$$a=U$$

$$b=\left( R -N_{\gamma}+ N_{\gamma}\times OR-OR\times U \right)$$

$$c=-OR\times R$$

$$\beta=\frac{-b\pm\sqrt{b^{2}-4ac}}{2a}$$

# **References**

1. Update on the E-2020 initiative of 21 malaria-eliminating countries: report and country briefs. Geneva: World Health Organization, 2018.

2. The Malaria Atlas Project (MAP) 2022 [cited 2022 September 06]. Available from: <https://malariaatlas.org/>.

3. World Population Prospects 2022 [cited 2022 September 22]. Available from: <https://population.un.org/wpp/Download/Standard/Population/>.

4. United Nations Department of Economic and Social Affairs/Statistics Division 2021 [cited 2022 April 05]. Available from: <https://unstats.un.org/unsd/methodology/m49/>.

5. The World Bank: data 2021 [cited 2022 April 05]. Available from: <https://data.worldbank.org/indicator/SP.RUR.TOTL.ZS?locations=ZG>.

6. WHO Global Health Observatory data repository “by theme” 2022 [cited 2022 September 06]. Available from: <https://www.who.int/data/gho>.

7. World malaria report 2021. Geneva: World Health Organization, 2021.

8. AIDSinfo: Global data on HIV epidemiology and response 2022 [cited 2022 September 06]. Available from: <http://aidsinfo.unaids.org/>.

9. Camponovo F, Bever CA, Galactionova K, Smith T, Penny MA. Incidence and admission rates for severe malaria and their impact on mortality in Africa. Malar J. 2017;16(1):1. Epub 2017/01/05. doi: 10.1186/s12936-016-1650-6. PubMed PMID: 28049519; PubMed Central PMCID: PMCPMC5209951.

10. WorldWide Antimalarial Resistance Network Falciparum Haematology Study Group. Haematological consequences of acute uncomplicated falciparum malaria: a WorldWide Antimalarial Resistance Network pooled analysis of individual patient data. BMC Med. 2022;20(1):85. Epub 20220307. doi: 10.1186/s12916-022-02265-9. PubMed PMID: 35249546.

11. Dellicour S, Tatem AJ, Guerra CA, Snow RW, ter Kuile FO. Quantifying the number of pregnancies at risk of malaria in 2007: a demographic study. PLoS Med. 2010;7(1):e1000221. Epub 2010/02/04. doi: 10.1371/journal.pmed.1000221. PubMed PMID: 20126256; PubMed Central PMCID: PMCPMC2811150.

12. Bearak J, Popinchalk A, Ganatra B, Moller AB, Tuncalp O, Beavin C, et al. Unintended pregnancy and abortion by income, region, and the legal status of abortion: estimates from a comprehensive model for 1990-2019. Lancet Glob Health. 2020;8(9):e1152-e61. Epub 2020/07/28. doi: 10.1016/S2214-109X(20)30315-6. PubMed PMID: 32710833.

13. Doumbe-Belisse P, Kopya E, Ngadjeu CS, Sonhafouo-Chiana N, Talipouo A, Djamouko-Djonkam L, et al. Urban malaria in sub-Saharan Africa: dynamic of the vectorial system and the entomological inoculation rate. Malar J. 2021;20(1):364. Epub 20210908. doi: 10.1186/s12936-021-03891-z. PubMed PMID: 34493280; PubMed Central PMCID: PMCPMC8424958.

14. Hay SI, Guerra CA, Tatem AJ, Atkinson PM, Snow RW. Urbanization, malaria transmission and disease burden in Africa. Nat Rev Microbiol. 2005;3(1):81-90. doi: 10.1038/nrmicro1069. PubMed PMID: 15608702; PubMed Central PMCID: PMCPMC3130901.

15. Robert V, Macintyre K, Keating J, Trape JF, Duchemin JB, Warren M, et al. Malaria transmission in urban sub-Saharan Africa. Am J Trop Med Hyg. 2003;68(2):169-76. PubMed PMID: 12641407.

16. Fagbamigbe AF, Kandala NB, Uthman AO. Demystifying the factors associated with rural-urban gaps in severe acute malnutrition among under-five children in low- and middle-income countries: a decomposition analysis. Sci Rep. 2020;10(1):11172. Epub 20200707. doi: 10.1038/s41598-020-67570-w. PubMed PMID: 32636405; PubMed Central PMCID: PMCPMC7341744.

17. USAID: The DHS Program STAT compiler 2022 [cited 2022 June 29]. Available from: <https://www.statcompiler.com/en/>.

18. Garcia-Calleja JM, Gouws E, Ghys PD. National population based HIV prevalence surveys in sub-Saharan Africa: results and implications for HIV and AIDS estimates. Sex Transm Infect. 2006;82 Suppl 3:iii64-70. doi: 10.1136/sti.2006.019901. PubMed PMID: 16735296; PubMed Central PMCID: PMCPMC2576729.

19. Jiwani SS, Gatica-Dominguez G, Crochemore-Silva I, Maiga A, Walton S, Hazel E, et al. Trends and inequalities in the nutritional status of adolescent girls and adult women in sub-Saharan Africa since 2000: a cross-sectional series study. BMJ Glob Health. 2020;5(10). doi: 10.1136/bmjgh-2020-002948. PubMed PMID: 33033052; PubMed Central PMCID: PMCPMC7545504.

20. Grant RL. Converting an odds ratio to a range of plausible relative risks for better communication of research findings. BMJ. 2014;348:f7450. Epub 20140124. doi: 10.1136/bmj.f7450. PubMed PMID: 24464277.
