# Supplementary material for: Characterisation of populations at risk of sub-optimal dosing of artemisinin-based combination therapy in Africa
Source: PLOS Glob Public Health. 2023 Dec 1;3(12):e0002059. doi: 10.1371/journal.pgph.0002059 (PMC10691722; doi:10.1371/journal.pgph.0002059)
Supplement: S2 Text — (DOCX) [file pgph.0002059.s002.docx]

**Characterisation of populations at risk of sub-optimal dosing of artemisinin-based combination therapy in Africa**

# **Systematic review** **to estimate failure rates of ACTs in sub-populations of interest**

Search for relevant studies was conducted on Epistemonikos [1]. Search terms were (falciparum malaria) AND (efficacy) AND (mefloquine OR lumefantrine OR amodiaquine OR piperaquine OR coartem OR artemether OR artemisinin OR ACT OR pyronaridine OR (combination therapy)). Last search was conducted on 08 September 2022; no time limit was set. Publications were eligible for inclusion if they were systematic reviews, reported individual patient data (IPD) meta-analysis and explored relationship between risk factors of interest and recrudescence; or reported any type of meta-analysis of recrudescence rate in a population of patients defined by the risk groups of interest (wasted children <5 years of age, pregnancy, PLHIV, hyperparasitaemia, overweight adults), in patients with uncomplicated falciparum malaria treated with ACTs, and followed-up at least 28 days.

Two independent reviewers (VC, KS) evaluated study eligibility and extracted data. Data extracted included information on treatment used and pooled estimate of the HR for recrudescence for each reported subgroup of interest. Where available, fixed-effect pooled estimates from meta-analyses’ Hazard Ratios (HR) were calculated by risk group of interest. Meta-analysis estimates and pooled estimates were presented in forest plots with heterogeneity measures (I^2^, τ^2^, ρ).

The systematic review identified five IPD meta-analyses [2-6] which provided HR estimates for children <5 years of age and for hyperparasitaemic patients (Fig A).

References

1. Epistemonikos database on best of evidence-based health care, information technologies and a network of experts to provide a unique tool for people making decisions concerning clinical or health-policy questions 2022 [cited 2022 September 08]. Available from: <https://www.epistemonikos.org/>.

2. Saito M, Mansoor R, Kennon K, Anvikar AR, Ashley EA, Chandramohan D, et al. Efficacy and tolerability of artemisinin-based and quinine-based treatments for uncomplicated falciparum malaria in pregnancy: a systematic review and individual patient data meta-analysis. Lancet Infect Dis. 2020;20(8):943-52. Epub 20200429. doi: 10.1016/S1473-3099(20)30064-5. PubMed PMID: 32530424; PubMed Central PMCID: PMCPMC7391007.

3. Worldwide Antimalarial Resistance Network A-L Dose Impact Study Group. The effect of dose on the antimalarial efficacy of artemether-lumefantrine: a systematic review and pooled analysis of individual patient data. Lancet Infect Dis. 2015;15(6):692-702. Epub 20150316. doi: 10.1016/S1473-3099(15)70024-1. PubMed PMID: 25788162; PubMed Central PMCID: PMCPMC4632191.

4. WorldWide Antimalarial Resistance Network AS-AQ Study Group. The effect of dosing strategies on the therapeutic efficacy of artesunate-amodiaquine for uncomplicated malaria: a meta-analysis of individual patient data. BMC Med. 2015;13:66. Epub 20150331. doi: 10.1186/s12916-015-0301-z. PubMed PMID: 25888957; PubMed Central PMCID: PMCPMC4411752.

5. WorldWide Antimalarial Resistance Network DP Study Group. The effect of dosing regimens on the antimalarial efficacy of dihydroartemisinin-piperaquine: a pooled analysis of individual patient data. PLoS Med. 2013;10(12):e1001564. Epub 20131203. doi: 10.1371/journal.pmed.1001564. PubMed PMID: 24311989; PubMed Central PMCID: PMCPMC3848996.

6. WorldWide Antimalarial Resistance Network Lumefantrine PK-PD Study Group. Artemether-lumefantrine treatment of uncomplicated Plasmodium falciparum malaria: a systematic review and meta-analysis of day 7 lumefantrine concentrations and therapeutic response using individual patient data. BMC Med. 2015;13:227. Epub 20150918. doi: 10.1186/s12916-015-0456-7. PubMed PMID: 26381375; PubMed Central PMCID: PMCPMC4574542.

### **Fig A. PRISMA flow diagram**

**Identification of studies**

Records removed *before screening*:

Broad synthesis (n = 4)

Structured summaries (n = 9)

Original studies (n = 678)

Records identified from:

Epistemonikos (n = 853)

**Identification**

Records excluded:

(n = 6 without full text access)

(n = 3 duplicates)

(n = 142 no outcome of interest reported)

Records screened:

(n = 162 systematic reviews)

**Screening**

Reports excluded:

Mixture of treatments examined or heterogeneity of study designs (n = 6)

Reports assessed for eligibility:

(n = 11)

Studies included in review:

(n = 5)

WWARN DP Study Group, 2013 (PubMed ID 24311989)

WWARN A-L Dose Impact Study Group, 2015 (PubMed ID 25788162)

WWARN AS-AQ Study Group, 2015 (PubMed ID 25888957)

WWARN PK/PD Study Group, 2015 (PubMed ID 26381375)

Saito M, 2020 (PubMed ID 32530424)

**Included**
