# Supplementary material for: Characterisation of populations at risk of sub-optimal dosing of artemisinin-based combination therapy in Africa
Source: PLOS Glob Public Health. 2023 Dec 1;3(12):e0002059. doi: 10.1371/journal.pgph.0002059 (PMC10691722; doi:10.1371/journal.pgph.0002059)
Supplement: S3 Text — (DOCX) [file pgph.0002059.s003.docx]

**Characterisation of populations at risk of sub-optimal dosing of artemisinin-based combination therapy in Africa**

### **Systematic review of the efficacy of artemisinin-based combination therapy (ACT) in adults in Africa with uncomplicated Plasmodium falciparum malaria who are overweight or obese**

**Methods**

MEDLINE, EMBASE, Global Health, EBSCO CINAHL, Scopus, Web of Science Core Collection, The Cochrane Library were searched without restrictions on language or publication date with a set of search terms described in Table S4. A librarian (ET) conducted the last search on 15 January 2018.

### **Table A. Search terms used in the systematic review**

| 1 | exp malaria/ | 14 | (artesunate and sulphadoxine and pyrimethamine).mp. |
| --- | --- | --- | --- |
| 2 | exp Plasmodium/ | 15 | 6 or 7 or 8 or 9 or 10 or 11 or 12 or 13 or 14 |
| 3 | malaria*.mp. | 16 | exp obesity/ |
| 4 | falciparum.mp. | 17 | exp body weight/ |
| 5 | 1 or 2 or 3 or 4 | 18 | Body Mass Index/ |
| 6 | artemisinin/ or artemisinin derivative/ | 19 | exp skinfold thickness/ |
| 7 | artemisinin*.mp. | 20 | waist hip ratio/ |
| 8 | Coartem.mp. | 21 | weight.ti. |
| 9 | artemether.mp. | 22 | fat.ti. |
| 10 | lumefantrin*.mp. | 23 | (overweight or "weight status" or bodyweight or "body mass" or fatness or "body weight").mp. |
| 11 | (artesunate and amodiaquine).mp. | 24 | obes*.mp. |
| 12 | (dihydroartemisinin and piperaquine).mp. | 25 | 16 or 17 or 18 or 19 or 20 or 21 or 22 or 23 or 24 |
| 13 | (artesunate and mefloquine).mp. | 26 | 5 and 15 and 25 |

Animal studies, case reports, cases series, systematic reviews and literature reviews were excluded from the search, as were patients with unconfirmed or asymptomatic parasitaemia, or with severe malaria. At the stage of full text screening two reviewers (AT, GI or MP) retained only manuscripts including participants with a body-weight above 70 kg, regardless of BMI, as height is seldom measured or reported. For the majority of studies, it was not possible to clearly ascertain at the stage of abstract screening whether weight was a factor considered in determining the efficacy of ACTs.

Primary outcome was PCR-adjusted treatment failure rate (recrudescence rate), as defined by WHO, on day 28 of treatment. Secondary outcomes were other measures of treatment failure including: PCR confirmed reinfection, recurrence, early treatment failure as well as outcomes recorded at day 42 or 63 of follow-up. Data extracted included study year, study site, study design, the study's inclusion and exclusion criteria, number of enrolled patients, treatment regimens, number of patients actually treated with each regimen, and reported outcomes. Treatment efficacy results were extracted whether presented separately for overweight or obese patients and patients considered to have normal weight on day 3 (early treatment failure), 7, 28, 42, or 63. For each reported outcome measure of treatment, difference in antimalarial treatment efficacy, such as Hazards Ratios and Odds Ratios with 95% confidence intervals and standard errors, were also extracted. The Newcastle-Ottawa Scale (NOS) for assessing the quality of nonrandomised studies [1] was used to evaluate evidence for each study by 2 independent reviewers (AT,MP).

**Results**

Using search terms as reported in Table A, we identified 554 unique articles; after abstract screening, 139 were selected for full-text screening (Fig A). Only 2 of these articles [2, 3] included relevant information and are described in this report.

The study by Gray *et al.* [2] is the only study identified which directly compares clinical outcomes in patient groups receiving AL defined by their BMI; BMI of 25kg/m^2^ was selected as the threshold for being overweight / obese. Surveillance data of 203 malaria cases, including 32 children below 16 years of age, with age range 1-88 years, reported to the CDC over a five-year period (May 2010 to April 2015), treated with artemether-lumefantrine was available. All cases were imported (US travellers or foreign visitors), with 61.1 % of the malaria infections acquired in West Africa, and the remaining infections acquired in East Africa, Central Africa, South Africa, the Caribbean, or South America. All patients had malaria confirmed by positive RDT, microscopy or PCR, except for one patient described as ‘clinically suspected’, diagnosed on clinical grounds. Outcome studied was defined as cure rate on day 7 and day 28, determined using the clinician’s assessment of malaria symptoms. BMI status and clinical outcomes were only available in a subset of patients (79 on day 7 and 69 on day 28), and it is unclear how many children or if the suspected malaria case was included in this subset. Upon clinical grounds alone, on day 7 and day 28, cure rates were not different between the groups ( Day 7: 39/40, 97.5%, 95% CI 86.8-99.9% in those who had a BMI above 25kg/m2 compared to 35/39, 89.7%; 95%CI 75.81- 97.1 in those with a BMI < 25kg/m2; Day 28: 38/39, 97.4%, 95%CI 86.5-99.9% in those with a BMI ≥ 25 compared to 28/30, 93.3%, 95%CI 77.9-99.2% in those with a BMI < 25).

The other study, by Toovey *et al.* [3] examined treatment outcomes of 123 adults with uncomplicated falciparum malaria, treated with artemether-lumefantrine (AL) between July 2001 and November 2003 at the occupational health centre in Mozambique. Expatriate workers (n=73) were classified as non-immune and Mozambican workers (n=50) were classified as semi-immune. Authors state that that they report treatment outcomes in subjects exceeding 65kg body weight but the minimum weight observed was not given; reported mean (SD, maximum) weight was 71kg (6.0, 95kg) and 81kg (11.7, 120kg) in semi-immune and non-immune groups, respectively. The case definition was the presence of a febrile illness together with either a positive rapid antigen test or positive Giemsa-stained peripheral blood smear for semi-immune group and the presence of either a positive smear or rapid histidine-rich protein II antigen test for Plasmodium falciparum, with no history of malaria in the preceding 4 weeks for non-immune subjects. The assessment of treatment failure did not fit strictly the WHO criteria of early treatment failure; non-immune patients were tested for parasitaemia and clinical symptoms on each day of treatment, whilst the 50 semi-immune patients were only assessed on the third day. None (0%, 0/50) of the semi-immune patients had treatment failure, whilst two non-immune (2.7%, 2/73) patients were still parasitaemic on day 3 of follow-up. The two patients who failed treatment weighed 88kg and 108kg.

### **Fig A. Study profile**

**Records identified** (n = 1476)

Ovid Medline (n = 245)

Ovid Embase (n = 436)

Ovid Global Health (n = 183)

Ebsco Cinahl (n = 11)

Scopus (n = 296)

Web of Science Core Collection (n = 196)

The Cochrane Library (n = 109)

**Records after de-duplication** (n = 554)

**Records excluded** (n = 415)

**Full-text articles assessed for eligibility** (n = 139)

**Exclusions** (n = 137)

Not uncomplicated malaria (n = 9)

Non-ACT treatments (n = 4)

No outcomes (n = 8)

Other design (n = 4)

Conference abstract (n = 23)

No relevant weight data (n = 89)

**Studies identified** (n = 2)

**Risk of bias assessment**

Quality of the data is considered to be fair (Table B), however none of the two identified studies assessed efficacy of ACTs in obese or overweight patients according to WHO guidelines.

### **Table B. Risk of bias assessment.**

The Newcastle-Ottawa Scale (NOS) for assessing the quality of nonrandomised studies was used. For assessment categories see footnotes.

| **RoB Criteria** | **Gray, 2015** | **Toovey, 2004** |
| --- | --- | --- |
| **Selection** | | |
| Representativeness of the exposed cohort^1^ | b | b |
| Selection of the non-exposed cohort^2^ | a | no non-exposure cohort |
| Ascertainment of exposure^3^ | a | a |
| Demonstration that outcome of interest was not present at start of study | Yes | Yes |
| **Comparability** | | |
| Comparability of cohorts on the basis of the design or analysis | No description | No description |
| **Outcome** | | |
| Assessment of outcome^4^ | Clinician evaluation | a |
| Was follow-up long enough for outcomes to occur | Yes (day 7, 28) | Yes (day 3) |
| Adequacy of follow up of cohorts^5^ | 61% and 66% missing | Complete (day 3) |

^1^ a) truly representative, b) somewhat representative, c) selected group of users d) no description;

^2^ a) drawn from the same community as the exposed cohort, b) drawn from a different source, c) no description of the derivation of the non-exposed cohort;

^3^ a) secure record, b) structured interview, c) written self-report, d) no description;

^4^ a) independent blind assessment, b) record linkage, c) self-report, d) no description;

^5^a) complete follow up, b) subjects lost to follow up unlikely to introduce bias, c) follow up rate and no description of those lost, d) no statement.

**Conclusion**

In this systematic review, not a single study was identified which assessed efficacy of ACTs in obese or overweight patients according to WHO guidelines

# **References**

1. Wells G, Shea B, O'Connell D, Welch V, Losos M, Tugwell P. The Newcastle-Ottawa Scale (NOS) for assessing the quality of nonrandomised studies in meta-analyses.: The Ottawa Hospital Research Institute; 2013 [cited 2022 July 12]. Available from: <http://www.ohri.ca/programs/clinical_epidemiology/oxford.asp>

2. Gray AM, Arguin PM, Hamed K. Surveillance for the safety and effectiveness of artemether-lumefantrine in patients with uncomplicated Plasmodium falciparum malaria in the USA: a descriptive analysis. Malar J. 2015;14:349. Epub 20150917. doi: 10.1186/s12936-015-0881-2. PubMed PMID: 26377423; PubMed Central PMCID: PMCPMC4573675.

3. Toovey S, Jamieson A. Treatment of uncomplicated falciparum malaria in non-immune and semi-immune individuals exceeding 65 kg body weight. S Afr Med J. 2004;94(6):443-4. PubMed PMID: 15250457.
