# Supplementary material for: Characterisation of populations at risk of sub-optimal dosing of artemisinin-based combination therapy in Africa
Source: PLOS Glob Public Health. 2023 Dec 1;3(12):e0002059. doi: 10.1371/journal.pgph.0002059 (PMC10691722; doi:10.1371/journal.pgph.0002059)
Supplement: S4 Text — (DOCX) [file pgph.0002059.s004.docx]

**Characterisation of populations at risk of sub-optimal dosing of artemisinin-based combination therapy in Africa**

# **Country-specific data**

Variables names and units correspond to those reported in Table B in S1 Text unless stated otherwise.

**Raw country data**

Levels of malaria endemicity were categorised as hypo-endemic (*Pf* rate in children age 2-9 years of age ≤10%); meso-endemic (*Pf* rate in children age 2-9 years of age 11-50%); hyperendemic (*Pf* rate in children age 2-9 years of age >50%) [1]. Distribution of malaria cases across age categories (<5 years old: prop_0_5; 5-10 years old: prop_5_10; 10-15 years old: prop_10_15; ≥15years old: prop_15_plus) was estimated using a model developed by Griffin *et al*. [2].

# **References**

1. WHO malaria terminology; updated in December 2019. Geneva: World Health Organization, 2016.

2. Griffin JT, Ferguson NM, Ghani AC. Estimates of the changing age-burden of Plasmodium falciparum malaria disease in sub-Saharan Africa. Nat Commun. 2014;5:3136. Epub 2014/02/13. doi: 10.1038/ncomms4136. PubMed PMID: 24518518; PubMed Central PMCID: PMCPMC3923296.

## **Table A. Raw malaria data per country**

| **Country** | **Region** | **Endemicity** | **prev** | **Confirmed RDT cases** | **Confirmed Micro cases** | **Confirmed cases** | **Severe** | **Hyper** | **prop _0_5** | **prop_ 5_10** | **prop_ 10_15** | **prop_ 15_plus** |
| --- | --- | --- | --- | --- | --- | --- | --- | --- | --- | --- | --- | --- |
| Angola | Central Africa | meso-endemicity | 0.23 | 4239539 | 2359788 | 6599327 | 296.1 | 0.102 | 0.23 | 0.23 | 0.15 | 0.39 |
| Benin | Western Africa | meso-endemicity | 0.36 | 2273685 | 242780 | 2516465 | 225.4 | 0.102 | 0.34 | 0.24 | 0.14 | 0.28 |
| Burkina Faso | Western Africa | meso-endemicity | 0.35 | 10519323 | 81017 | 10600340 | 427.2 | 0.102 | 0.34 | 0.24 | 0.14 | 0.28 |
| Burundi | Eastern Africa | meso-endemicity | 0.29 | 3963662 | 756441 | 4720103 | 337.5 | 0.102 | 0.28 | 0.24 | 0.15 | 0.33 |
| Cameroon | Central Africa | meso-endemicity | 0.22 | 1933546 | 956647 | 2890193 | 240.1 | 0.102 | 0.23 | 0.22 | 0.15 | 0.40 |
| Central African Republic | Central Africa | meso-endemicity | 0.33 | 1563228 | 177742 | 1740970 | 167.9 | 0.102 | 0.31 | 0.24 | 0.14 | 0.30 |
| Chad | Central Africa | meso-endemicity | 0.14 | 1350378 | 193816 | 1544194 | 146.2 | 0.102 | 0.16 | 0.19 | 0.16 | 0.48 |
| Congo | Central Africa | meso-endemicity | 0.21 | 36190 | 55348 | 91538 | 74.9 | 0.102 | 0.21 | 0.22 | 0.16 | 0.41 |
| Côte d'Ivoire | Western Africa | meso-endemicity | 0.28 | 4048013 | 932627 | 4980640 | 124.1 | 0.102 | 0.27 | 0.23 | 0.15 | 0.34 |
| Democratic Republic of the Congo | Central Africa | meso-endemicity | 0.36 | 21376223 | 1214424 | 22590647 | 435.1 | 0.102 | 0.34 | 0.24 | 0.14 | 0.28 |
| Djibouti | Eastern Africa | hypo-endemicity | 0.01 | 73535 | 11633 | 85168 | 37.4 | 0.102 | 0.08 | 0.12 | 0.13 | 0.68 |
| Equatorial Guinea | Central Africa | meso-endemicity | 0.24 | 11117* | 14787* | 25904* | 1.8& | 0.102 | 0.24 | 0.23 | 0.15 | 0.38 |
| Eritrea | Eastern Africa | hypo-endemicity | 0.01 | 56105 | 17936 | 74041 | 3.2 | 0.102 | 0.08 | 0.12 | 0.13 | 0.68 |
| Ethiopia | Eastern Africa | hypo-endemicity | 0.02 | -- | 1743755 | 1743755 | 2.3 | 0.102 | 0.08 | 0.13 | 0.13 | 0.66 |
| Gabon | Central Africa | meso-endemicity | 0.21 | 20310 | 33349 | 53659 | 121.1 | 0.102 | 0.22 | 0.22 | 0.16 | 0.41 |
| Gambia | Western Africa | hypo-endemicity | 0.04 | 53575 | 22226 | 75801 | 97.8 | 0.102 | 0.09 | 0.14 | 0.14 | 0.63 |
| Ghana | Western Africa | meso-endemicity | 0.18 | 4046554 | 1401009 | 5447563 | 106.7 | 0.102 | 0.19 | 0.21 | 0.16 | 0.45 |
| Guinea | Western Africa | meso-endemicity | 0.29 | 1891408 | 117568 | 2008976 | 115.6 | 0.102 | 0.28 | 0.24 | 0.15 | 0.33 |
| Guinea-Bissau | Western Africa | hypo-endemicity | 0.05 | 115232* | 45675* | 160907* | 231.5 | 0.102 | 0.10 | 0.15 | 0.14 | 0.60 |
| Kenya | Eastern Africa | hypo-endemicity | 0.03 | 2012522 | 1646648 | 3659170 | 13.1 | 0.102 | 0.09 | 0.13 | 0.14 | 0.64 |
| Liberia | Western Africa | meso-endemicity | 0.4 | 590187* | 325658* | 915845* | 696.9 | 0.102 | 0.38 | 0.24 | 0.13 | 0.25 |
| Madagascar | Eastern Africa | hypo-endemicity | 0.06 | 1945396 | 5075 | 1950471 | 26.4 | 0.102 | 0.11 | 0.15 | 0.15 | 0.59 |
| Malawi | Eastern Africa | meso-endemicity | 0.18 | 7057864 | 81201 | 7139065 | 339.2 | 0.102 | 0.20 | 0.21 | 0.16 | 0.44 |
| Mali | Western Africa | meso-endemicity | 0.21 | 2124511 | 541755 | 2666266 | 179.7 | 0.102 | 0.21 | 0.22 | 0.16 | 0.41 |
| Mauritania | Western Africa | hypo-endemicity | 0.05 | 12425 | -- | 12425 | 5.4 | 0.102 | 0.10 | 0.15 | 0.14 | 0.60 |
| Mozambique | Eastern Africa | meso-endemicity | 0.29 | 10845525 | 473160 | 11318685 | 156.3 | 0.102 | 0.28 | 0.23 | 0.15 | 0.34 |
| Namibia | Southern Africa | hypo-endemicity | 0.007 | 13636 | 168 | 13804 | 30.9 | 0.102 | 0.07 | 0.12 | 0.13 | 0.69 |
| Niger | Western Africa | meso-endemicity | 0.24 | 4154337 | 223601 | 4377938 | 180.6 | 0.102 | 0.24 | 0.23 | 0.15 | 0.39 |
| Nigeria | Western Africa | meso-endemicity | 0.28 | 16013077 | 2312163 | 18325240 | 44.8 | 0.102 | 0.27 | 0.23 | 0.15 | 0.34 |
| Rwanda | Eastern Africa | hypo-endemicity | 0.02 | 1549912 | 493480 | 2043392 | 52.9 | 0.102 | 0.09 | 0.13 | 0.14 | 0.65 |
| Sao Tome and Principe | Central Africa | hypo-endemicity | 0.01 | 400 | 1544 | 1944 | 0.0 | 0.102 | 0.07 | 0.12 | 0.13 | 0.68 |
| Senegal | Western Africa | hypo-endemicity | 0.04 | 441432 | 3881 | 445313 | 38.5 | 0.102 | 0.10 | 0.14 | 0.14 | 0.62 |
| Sierra Leone | Western Africa | meso-endemicity | 0.33 | 654005 | 71001 | 725006 | 618 | 0.102 | 0.32 | 0.24 | 0.14 | 0.30 |
| Somalia | Eastern Africa | hypo-endemicity | 0.07 | 27333 | -- | 27333 | 1.5 | 0.102 | 0.11 | 0.16 | 0.15 | 0.58 |
| Somalia | Eastern Africa | hypo-endemicity | 0.07 | 27333 | -- | 27333 | 1.5 | 0.102 | 0.11 | 0.16 | 0.15 | 0.58 |
| South Sudan | Eastern Africa | meso-endemicity | 0.28 | 192095 | 16535 | 208630 | 0.57& | 0.102 | 0.27 | 0.23 | 0.15 | 0.35 |
| Sudan | Northern Africa | hypo-endemicity | 0.04 | 435553 | 1262841 | 1698394 | 23.0 | 0.102 | 0.10 | 0.14 | 0.14 | 0.62 |
| Togo | Western Africa | meso-endemicity | 0.2 | 2136565* | 269526* | 2406091* | 224.7 | 0.102 | 0.20 | 0.21 | 0.16 | 0.43 |
| Uganda | Eastern Africa | meso-endemicity | 0.2 | 12548724 | 1647933 | 14196657 | 207.3 | 0.102 | 0.20 | 0.21 | 0.16 | 0.42 |
| United Republic of Tanzania | Eastern Africa | hypo-endemicity | 0.08 | 5681861 | 296052 | 5977913 | 126.4 | 0.102 | 0.12 | 0.17 | 0.15 | 0.56 |
| Zambia | Eastern Africa | meso-endemicity | 0.15 | 7992924 | 128291 | 8121215 | 271.7 | 0.102 | 0.17 | 0.20 | 0.16 | 0.48 |
| Zimbabwe | Eastern Africa | hypo-endemicity | 0.04 | 447381 | -- | 447381 | 30.1 | 0.102 | 0.09 | 0.14 | 0.14 | 0.62 |

* malaria data available for 2019 only

& data on severe cases not available at country level; instead an estimate based on the median proportion of severe cases in the region was used

## **Table B. Raw population (in 1000) data per country**

| **Country** | **Population** | **Rural_pop proportion** | **M0_14** | **F0_14** | **M18plus** | **F18plus** | **M15plus** | **F15plus** | **Adults 15plus** | **F15to49** |
| --- | --- | --- | --- | --- | --- | --- | --- | --- | --- | --- |
| Angola | 33428.486 | 33.2 | 7608.702 | 7536.609 | 7833.7195 | 8298.707 | 8908.7945 | 9374.3805 | 18283.175 | 7708.0285 |
| Benin | 12643.123 | 51.6 | 2736.7795 | 2663.435 | 3179.169 | 3246.5985 | 3593.9965 | 3648.912 | 7242.9085 | 2967.2585 |
| Burkina Faso | 21522.626 | 69.4 | 4857.067 | 4707.0545 | 5130.1995 | 5385.392 | 5861.5345 | 6096.97 | 11958.504 | 5036.092 |
| Burundi | 12220.227 | 86.3 | 2869.0185 | 2840.3055 | 2799.3075 | 2915.9525 | 3197.5805 | 3313.322 | 6510.9025 | 2801.594 |
| Cameroon | 26491.087 | 42.4 | 5683.9615 | 5607.908 | 6653.8375 | 6803.5205 | 7526.6645 | 7672.5525 | 15199.217 | 6406.259 |
| Central African Republic | 5343.02 | 57.8 | 1300.002 | 1275.7845 | 1167.479 | 1193.7365 | 1372.078 | 1395.1555 | 2767.2335 | 1146.7535 |
| Chad | 16644.701 | 76.5 | 4036.79 | 3922.162 | 3758.611 | 3821.7815 | 4318.2455 | 4367.504 | 8685.7495 | 3683.4875 |
| Congo | 5702.174 | 32.2 | 1198.1095 | 1178.097 | 1470.6165 | 1499.644 | 1649.443 | 1676.5245 | 3325.9675 | 1364.498 |
| Côte d'Ivoire | 26811.79 | 48.3 | 5685.268 | 5605.2775 | 6923.403 | 6749.008 | 7862.3535 | 7658.8915 | 15521.245 | 6531.092 |
| Democratic Republic of the Congo | 92853.164 | 54.4 | 21621.669 | 21549.416 | 21465.169 | 22252.352 | 24444.309 | 25237.77 | 49682.079 | 20583.703 |
| Djibouti | 1090.156 | 21.9 | 171.9525 | 168.079 | 334.709 | 346.3705 | 369.518 | 380.6065 | 750.1245 | 297.2115 |
| Equatorial Guinea | 1596.049 | 26.9 | 312.666 | 307.492 | 489.805 | 405.0585 | 531.4865 | 444.404 | 975.8905 | 356.102 |
| Eritrea | 3555.868 | 58.7 | 725.478 | 711.98 | 895.3905 | 959.5415 | 1028.035 | 1090.375 | 2118.41 | 858.0685 |
| Ethiopia | 117190.91 | 78.3 | 24084.32 | 23159.076 | 30720.147 | 31137.139 | 34822.23 | 35125.285 | 69947.515 | 28875.771 |
| Gabon | 2292.573 | 9.9 | 421.052 | 416.3645 | 683.373 | 643.2425 | 747.7705 | 707.386 | 1455.1565 | 567.863 |
| Gambia | 2573.995 | 37.4 | 571.316 | 559.3215 | 620.048 | 647.6705 | 708.717 | 734.6405 | 1443.3575 | 611.2775 |
| Ghana | 32180.401 | 42.7 | 6120.742 | 5973.172 | 8915.929 | 9161.169 | 9930.573 | 10155.915 | 20086.487 | 8122.409 |
| Guinea | 13205.153 | 63.1 | 2824.452 | 2736.153 | 3246.9645 | 3517.708 | 3693.5825 | 3950.965 | 7644.5475 | 3187.8315 |
| Guinea-Bissau | 2015.828 | 55.8 | 418.109 | 408.0425 | 508.111 | 545.7665 | 576.497 | 613.1795 | 1189.6765 | 505.504 |
| Kenya | 51985.78 | 72.0 | 10162.324 | 10098.102 | 13826.978 | 14303.77 | 15618.374 | 16106.98 | 31725.355 | 13388.164 |
| Liberia | 5087.584 | 47.9 | 1067.634 | 1041.505 | 1287.9895 | 1341.9855 | 1463.82 | 1514.6255 | 2978.4455 | 1229.0255 |
| Madagascar | 28225.177 | 61.5 | 5674.8495 | 5536.0935 | 7540.5625 | 7621.4255 | 8473.575 | 8540.6585 | 17014.233 | 6946.3205 |
| Malawi | 19377.061 | 82.6 | 4231.8355 | 4241.313 | 4490.43 | 5003.474 | 5186.9225 | 5716.9905 | 10903.913 | 4862.6335 |
| Mali | 21224.04 | 56.1 | 5109.563 | 4994.7755 | 4868.831 | 4790.708 | 5608.776 | 5510.9255 | 11119.701 | 4660.533 |
| Mauritania | 4498.604 | 44.7 | 964.129 | 942.2035 | 1088.6515 | 1201.597 | 1238.289 | 1353.982 | 2592.271 | 1104.934 |
| Mozambique | 31178.239 | 62.9 | 6860.8105 | 6815.0475 | 7382.199 | 8016.017 | 8432.489 | 9069.892 | 17502.381 | 7481.792 |
| Namibia | 2489.098 | 48.0 | 448.3885 | 452.7555 | 683.01 | 762.4625 | 753.272 | 834.682 | 1587.954 | 652.1315 |
| Niger | 24333.639 | 83.4 | 6052.39 | 5858.5495 | 5457.4565 | 5327.968 | 6289.98 | 6132.719 | 12422.699 | 5075.045 |
| Nigeria | 208327.4 | 48.0 | 45967.224 | 44659.122 | 52238.421 | 51642.363 | 59275.95 | 58425.108 | 117701.06 | 47915.558 |
| Rwanda | 13146.362 | 82.6 | 2608.0125 | 2562.039 | 3366.272 | 3711.0425 | 3815.132 | 4161.178 | 7976.31 | 3400.923 |
| Sao Tome and Principe | 218.641 | 25.6 | 44.5465 | 44.252 | 57.13 | 57.5995 | 64.566 | 65.2765 | 129.8425 | 52.254 |
| Senegal | 16436.12 | 51.9 | 3499.205 | 3414.668 | 4030.614 | 4408.2375 | 4577.7205 | 4944.5265 | 9522.247 | 4027.1715 |
| Sierra Leone | 8233.97 | 57.1 | 1655.6585 | 1620.241 | 2188.781 | 2214.9335 | 2469.1095 | 2488.9615 | 4958.071 | 2024.469 |
| Somalia | 16537.016 | 53.9 | 3970.6505 | 3857.925 | 3755.866 | 3838.999 | 4319.8205 | 4388.6195 | 8708.44 | 3629.2915 |
| South Sudan | 10606.227 | 79.8 | 2444.0325 | 2381.538 | 2399.134 | 2583.5965 | 2804.039 | 2976.6175 | 5780.6565 | 2369.571 |
| Sudan | 44440.486 | 64.7 | 9273.9525 | 9025.5965 | 11543.599 | 11839.948 | 12935.481 | 13205.456 | 26140.937 | 10652.458 |
| Togo | 8442.58 | 57.2 | 1725.36 | 1693.7015 | 2243.645 | 2238.0065 | 2517.7025 | 2505.816 | 5023.5185 | 2037.0865 |
| Uganda | 44404.611 | 75.0 | 10203.319 | 10076.314 | 10132.986 | 10709.999 | 11769.771 | 12355.207 | 24124.978 | 10693.337 |
| United Republic of Tanzania | 61704.518 | 64.8 | 13667.434 | 13406.888 | 14699.709 | 15737.48 | 16808.019 | 17822.176 | 34630.196 | 14782.876 |
| Zambia | 18927.715 | 55.4 | 4131.891 | 4135.8055 | 4564.096 | 4803.12 | 5206.7225 | 5453.296 | 10660.019 | 4689.577 |
| Zimbabwe | 15669.666 | 67.8 | 3214.765 | 3234.256 | 3644.5475 | 4495.1675 | 4170.4545 | 5050.1905 | 9220.645 | 4228.3835 |

## **Table C. Raw fertility, nutrition and HIV data per country**

| **Country** | **CBR** | **TFR** | **AR** | **U5 wasted** | **Overwt Adult M** | **Overwt Adult F** | **HIV 0_14** | **HIV 15M** | **HIV 15F** | **HAART** |
| --- | --- | --- | --- | --- | --- | --- | --- | --- | --- | --- |
| Angola | 39.3 | 5.4 | 32 | 4.9 | 19.5 | 34.9 | 39000 | 100000 | 190000 | 111188 |
| Benin | 37.1 | 5.0 | 32 | 5 | 21.2 | 37.3 | 8900 | 23000 | 43000 | 52497 |
| Burkina Faso | 36.1 | 4.9 | 32 | 8.1 | 16.1 | 29.2 | 16000 | 32000 | 49000 | 70156 |
| Burundi | 35.6 | 5.2 | 35 | 4.8 | 13.9 | 30.1 | 9700 | 29000 | 44000 | 73033 |
| Cameroon | 35.5 | 4.5 | 32 | 4.3 | 25.2 | 41.7 | 35000 | 150000 | 310000 | 367871 |
| Central African Republic | 42.2 | 6.0 | 32 | 5.2 | 18.3 | 33.2 | 5800 | 31000 | 51000 | 50805 |
| Chad | 43.8 | 6.3 | 32 | 13.9 | 16.0 | 29.8 | 11000 | 38000 | 63000 | 77017 |
| Congo | 31.2 | 4.2 | 32 | 8.2 | 24.2 | 37.4 | 11000 | 31000 | 72000 | 27371 |
| Côte d'Ivoire | 34.2 | 4.5 | 32 | 6.1 | 24.5 | 39.5 | 21000 | 120000 | 240000 | 280848 |
| Democratic Republic of the Congo | 42.3 | 6.2 | 32 | 6.4 | 18.0 | 32.0 | 71000 | 120000 | 310000 | 376617 |
| Djibouti | 22.5 | 2.8 | 35 | 21.5 | 32.3 | 44.6 | <1000 | 2700 | 3500 | 2063 |
| Equatorial Guinea | 31.2 | 4.3 | 32 | 3.1 | 18.7 | 35.5 | 3500 | 29000 | 35000 | 25703 |
| Eritrea | 28.7 | 3.9 | 35 | 14.6 | 14.7 | 28.3 | <1000 | 4700 | 7100 | 9078 |
| Ethiopia | 32.8 | 4.2 | 35 | 7.2 | 13.4 | 28.0 | 44000 | 220000 | 360000 | 483127 |
| Gabon | 27.7 | 3.5 | 32 | 3.4 | 33.7 | 46.4 | 2900 | 13000 | 30000 | 24208 |
| Gambia | 33.7 | 4.8 | 32 | 5.1 | 24.5 | 38.9 | 2200 | 9500 | 15000 | 8286 |
| Ghana | 28.0 | 3.6 | 32 | 6.8 | 22.1 | 41.0 | 29000 | 100000 | 220000 | 208811 |
| Guinea | 34.9 | 4.5 | 32 | 9.2 | 18.7 | 34.2 | 9700 | 36000 | 68000 | 55993 |
| Guinea-Bissau | 31.6 | 4.1 | 32 | 5.1 | 22.2 | 37.2 | 3500 | 12000 | 22000 | 21967 |
| Kenya | 28.0 | 3.4 | 35 | 4.2 | 16.1 | 34.3 | 82000 | 480000 | 870000 | 1229955 |
| Liberia | 31.7 | 4.2 | 32 | 3.4 | 23.2 | 38.3 | 2800 | 12000 | 20000 | 18515 |
| Madagascar | 31.3 | 3.9 | 35 | 6.4 | 17.7 | 29.6 | 2100 | 21000 | 19000 | 5885 |
| Malawi | 33.2 | 4.0 | 35 | 0.6 | 14.8 | 31.5 | 62000 | 330000 | 600000 | 853032 |
| Mali | 42.0 | 6.0 | 32 | 9.3 | 20.3 | 35.1 | 14000 | 37000 | 59000 | 56901 |
| Mauritania | 33.4 | 4.5 | 32 | 11.5 | 26.0 | 42.3 | <1000 | 4200 | 3700 | 3384 |
| Mozambique | 37.0 | 4.7 | 35 | 4.4 | 18.0 | 33.6 | 130000 | 710000 | 1200000 | 1402900 |
| Namibia | 27.9 | 3.3 | 30 | 7.1 | 27.2 | 51.9 | 8400 | 71000 | 130000 | 181696 |
| Niger | 45.6 | 6.9 | 32 | 9.8 | 14.6 | 29.7 | 3200 | 12000 | 17000 | 21330 |
| Nigeria | 37.5 | 5.3 | 32 | 6.5 | 21.7 | 36.1 | 130000 | 650000 | 960000 | 1492151 |
| Rwanda | 30.3 | 3.9 | 35 | 1.1 | 15.6 | 33.5 | 12000 | 80000 | 130000 | 204711 |
| Sao Tome and Principe | 28.4 | 3.9 | 32 | 4.1 | 28.2 | 41.6 | .. | <500 | <500 | 871 |
| Senegal | 33.0 | 4.5 | 32 | 8.1 | 19.5 | 35.9 | 3900 | 14000 | 21000 | 30431 |
| Sierra Leone | 31.9 | 4.1 | 32 | 5.4 | 19.1 | 36.0 | 11000 | 26000 | 42000 | 38003 |
| Somalia | 44.0 | 6.4 | 35 | 14.3 | 20.3 | 35.9 | 1000 | 3800 | 3900 | 3696 |
| South Sudan | 29.2 | 4.5 | 35 | 22.7 | 19.7** | 36.1** | 16000 | 63000 | 100000 | 42447 |
| Sudan | 34.2 | 4.5 | 46 | 16.3 | 19.7** | 36.1** | 4100 | 23000 | 21000 | 12155 |
| Togo | 32.3 | 4.3 | 32 | 5.7 | 19.6 | 35.8 | 9700 | 36000 | 66000 | 80160 |
| Uganda | 37.3 | 4.7 | 35 | 3.5 | 13.7 | 30.9 | 98000 | 490000 | 820000 | 1279427 |
| United Republic of Tanzania | 36.7 | 4.8 | 35 | 3.5 | 19.6 | 35.5 | 110000 | 610000 | 1000000 | 1422782 |
| Zambia | 35.0 | 4.4 | 35 | 4.2 | 19.0 | 36.3 | 82000 | 520000 | 850000 | 1179031 |
| Zimbabwe | 31.0 | 3.5 | 35 | 2.9 | 22.2 | 52.8 | 79000 | 470000 | 720000 | 1184901 |

% latest data available in 2020 (Rwanda, Nigeria, Gambia) - in 2019 (Guinea-Bissau, Ethiopia, Chad, Burundi, Liberia, Sierra Leone, Mali, Malawi, Senegal, CAR, Niger, Burkina Faso, Zimbabwe)

% latest data available in 2018 (Guinea, Benin, Zambia, Cameroon, Tanzania, Mauritania, Madagascar) - in 2017 (Ghana, Togo, DRC) - in 2016 (Côte d'Ivoire, Uganda) - in 2015 (Mozambique, Angola)

% latest data available in 2014 (Kenya, Congo, Sudan) - in 2013 (Namibia) - in 2012 (Gabon, Djibouti) - in 2011 (Equatorial Guinea) - in 2010 (South Sudan, Eritrea) -in 2009 (Somalia)

**data on overweight adults available for Sudan (until 2011) and reported as similar for both Sudan and South Sudan in this spreadsheet

## **Table D: Extracted country-level variables with reported lower and upper limits**

## Only eight country-level variables had reported lower and upper limits.

| **Country** | **AR** | **U5 wasted** | **Overwt Adult M** | **Overwt Adult F** | **HIV 0_14** | **HIV 15M** | **HIV 15F** | **prev** |
| --- | --- | --- | --- | --- | --- | --- | --- | --- |
| Angola | 32 [21-46] | 4.9 [4.3-5.6] | 19.5 [12.4-27.3] | 34.9 [27.0-43.6] | 39000 [31000-49000] | 100000 [87000-130000] | 190000 [160000-230000] | 0.23 [0.15-0.35] |
| Benin | 32 [25-39] | 5 [4.5-5.4] | 21.2 [15.5-27.7] | 37.3 [31.4-43.4] | 8900 [7000-12000] | 23000 [21000-29000] | 43000 [37000-53000] | 0.36 [0.26-0.46] |
| Burkina Faso | 32 [25-39] | 8.1 [7.6-8.5] | 16.1 [11.1-21.7] | 29.2 [23.5-35.1] | 16000 [12000-21000] | 32000 [28000-37000] | 49000 [40000-56000] | 0.35 [0.17-0.53] |
| Burundi | 35 [30-40] | 4.8 [4.3-5.2] | 13.9 [9.1-19.7] | 30.1 [23.8-36.7] | 9700 [7300-12000] | 29000 [26000-33000] | 44000 [39000-50000] | 0.29 [0.21-0.39] |
| Cameroon | 32 [21-46] | 4.3 [3.4-5.4] | 25.2 [19.2-32.0] | 41.7 [35.6-47.9] | 35000 [27000-41000] | 150000 [140000-160000] | 310000 [290000-340000] | 0.22 [0.14-0.31] |
| Central African Republic | 32 [21-46] | 5.2 [4.7-5.8] | 18.3 [11.9-25.8] | 33.2 [35.9-41.4] | 5800 [4500-7800] | 31000 [26000-39000] | 51000 [42000-61000] | 0.33 [0.13-0.63] |
| Chad | 32 [21-46] | 13.9 [13.2-14.7] | 16.0 [10.8-22.1] | 29.8 [24.0-36.0] | 11000 [7600-15000] | 38000 [32000-46000] | 63000 [51000-75000] | 0.14 [0.07-0.23] |
| Congo | 32 [21-46] | 8.2 [7.1-9.4] | 24.2 [18.2-31.3] | 37.4 [29.8-45.2] | 11000 [7600-15000] | 31000 [22000-46000] | 72000 [50000-110000] | 0.21 [0.13-0.34] |
| Côte d'Ivoire | 32 [25-39] | 6.1 [5.5-6.7] | 24.5 [11.5-25.3] | 39.5 [33.2-46.0] | 21000 [16000-27000] | 120000 [110000-140000] | 240000 [210000-270000] | 0.28 [0.13-0.52] |
| Democratic Republic of the Congo | 32 [21-46] | 6.4 [5.7-7.3] | 18.0 [11.5-25.3] | 32.0 [25.1-39.5] | 71000 [56000-85000] | 120000 [110000-140000] | 310000 [270000-360000] | 0.36 [0.26-0.44] |
| Djibouti | 35 [30-40] | 21.5 [19.5-23.6] | 32.3 [23.7-41.3] | 44.6 [36.7-52.7] | <1000 [<500-<1000] | 2700 [2000-3600] | 3500 [2600-4600] | 0.01 [0.01-0.01] |
| Equatorial Guinea | 32 [21-46] | 3.1 [--] | 18.7 [11.8-26.6] | 35.5 [27.3-43.7] | 3500 [2600-4900] | 29000 [22000-41000] | 35000 [27000-48000] | 0.24 [0.10-0.41] |
| Eritrea | 35 [30-40] | 14.6 [13.6-15.6] | 14.7 [9.9-20.6] | 28.3 [22.5-34.5] | <1000 [<500-1000] | 4700 [3600-6000] | 7100 [5400-9300] | 0.01 [0.01-0.01] |
| Ethiopia | 35 [30-40] | 7.2 [--] | 13.4 [8.7-18.9] | 28.0 [22.2-34.1] | 44000 [29000-62000] | 220000 [180000-260000] | 360000 [290000-440000] | 0.02 [0.01-0.03] |
| Gabon | 32 [21-46] | 3.4 [2.7-4.3] | 33.7 [24.0-43.8] | 46.4 [38.0-55.0] | 2900 [2100-3700] | 13000 [11000-16000] | 30000 [25000-37000] | 0.21 [0.09-0.41] |
| Gambia | 32 [25-39] | 5.1 [--] | 24.5 [18.3-31.7] | 38.9 [32.5-45.5] | 2200 [1600-2900] | 9500 [7500-13000] | 15000 [12000-20000] | 0.04 [0.03-0.05] |
| Ghana | 32 [25-39] | 6.8 [6.1-7.6] | 22.1 [16.9-28.1] | 41.0 [35.2-46.8] | 29000 [23000-35000] | 100000 [89000-120000] | 220000 [190000-250000] | 0.18 [0.14-0.21] |
| Guinea | 32 [25-39] | 9.2 [8.1-10.5] | 18.7 [13.4-25.1] | 34.2 [28.2-40.4] | 9700 [8200-11000] | 36000 [33000-39000] | 68000 [64000-73000] | 0.29 [0.15-0.46] |
| Guinea-Bissau | 32 [25-39] | 5.1 [4.5-5.8] | 22.2 [15.9-29.6] | 37.2 [30.7-44.1] | 3500 [2900-4100] | 12000 [10000-14000] | 22000 [20000-24000] | 0.05 [0.01-0.14] |
| Kenya | 35 [30-40] | 4.2 [3.8-4.6] | 16.1 [11.0-22.0] | 34.3 [28.5-40.3] | 82000 [67000-100000] | 480000 [420000-570000] | 870000 [750000-1000000] | 0.03 [0.02-0.05] |
| Liberia | 32 [25-39] | 3.4 [--] | 23.2 [17.2-29.7] | 38.3 [32.1-44.6] | 2800 [2100-3700] | 12000 [9800-15000] | 20000 [17000-24000] | 0.40 [0.20-0.64] |
| Madagascar | 35 [30-40] | 6.4 [5.9-7.0] | 17.7 [12.3-24.2] | 29.6 [23.8-35.9] | 2100 [1700-2700] | 21000 [17000-28000] | 19000 [15000-24000] | 0.06 [0.04-0.09] |
| Malawi | 35 [30-40] | 0.6 [0.4-1.0] | 14.8 [9.8-20.7] | 31.5 [25.8-37.5] | 62000 [52000-71000] | 330000 [310000-360000] | 600000 [560000-650000] | 0.18 [0.08-0.31] |
| Mali | 32 [25-39] | 9.3 [8.6-10.1] | 20.3 [14.6-26.9] | 35.1 [29.1-41.4] | 14000 [11000-18000] | 37000 [30000-45000] | 59000 [49000-72000] | 0.21 [0.13-0.33] |
| Mauritania | 32 [25-39] | 11.5 [10.8-12.2] | 26.0 [19.2-33.4] | 42.3 [35.7-49.3] | <1000 [<1000-<1000] | 4200 [3400-5300] | 3700 [3100-4600] | 0.05 [0.04-0.08] |
| Mozambique | 35 [30-40] | 4.4 [3.8-5.1] | 18.0 [12.7-24.1] | 33.6 [27.9-39.8] | 130000 [100000-170000] | 710000 [570000-880000] | 1200000 [990000-11500000] | 0.29 [0.20-0.40] |
| Namibia | 30 [15-58] | 7.1 [6.1-8.4] | 27.2 [20.2-34.6] | 51.9 [45.0-58.8] | 8400 [7300-9300] | 71000 [66000-80000] | 130000 [120000-140000] | 0.007 [0.003-0.01] |
| Niger | 32 [25-39] | 9.8 [8.6-11.1] | 14.6 [9.8-20.6] | 29.7 [23.9-35.9] | 3200 [2700-3700] | 12000 [10000-13000] | 17000 [15000-19000] | 0.24 [0.11-0.41] |
| Nigeria | 32 [25-39] | 6.5 [6.0-7.0] | 21.7 [16.5-27.2] | 36.1 [30.7-41.8] | 130000 [85000-190000] | 650000 [490000-860000] | 960000 [720000-1300000] | 0.28 [0.20-0.37] |
| Rwanda | 35 [30-40] | 1.1 [--] | 15.6 [10.7-21.1] | 33.5 [27.6-39.5] | 12000 [9500-15000] | 80000 [74000-91000] | 130000 [120000-150000] | 0.02 [0.02-0.03] |
| Sao Tome and Principe | 32 [21-46] | 4.1 [3.1-5.4] | 28.2 [21.4-35.5] | 41.6 [34.8-48.6] | .. | <500 [<200-<500] | <500 [<500-<500] | 0.01 [0.01-0.01] |
| Senegal | 32 [25-39] | 8.1 [7.5-8.9] | 19.5 [14.1-25.6] | 35.9 [29.8-42.2] | 3900 [3300-4600] | 14000 [13000-16000] | 21000 [19000-24000] | 0.04 [0.03-0.06] |
| Sierra Leone | 32 [25-39] | 5.4 [4.7-6.2] | 19.1 [13.7-25.1] | 36.0 [29.9-42.1] | 11000 [9000-14000] | 26000 [23000-30000] | 42000 [37000-48000] | 0.33 [0.15-0.60] |
| Somalia | 35 [30-40] | 14.3 [12.2-16.7] | 20.3 [14.1-27.1] | 35.9 [28.9-43.1] | 1000 [<1000-1200] | 3800 [3300-4300] | 3900 [3400-4400] | 0.07 [0.05-0.10] |

**Table D. cont.-**

| South Sudan | 35 [30-40] | 22.7 [21.1-24.5] | 19.7 [13.9-26.4] | 36.1 [29.7-43.0] | 16000 [11000-21000] | 63000 [48000-79000] | 100000 [78000-130000] | 0.28 [0.12-0.43] |
| --- | --- | --- | --- | --- | --- | --- | --- | --- |
| Sudan | 46 [28-72] | 16.3 [15.2-17.4] | 19.7 [13.9-26.4] | 36.1 [29.7-43.0] | 4100 [3200-4800] | 23000 [20000-28000] | 21000 [18000-25000] | 0.04 [0.02-0.08] |
| Togo | 32 [25-39] | 5.7 [5.0-6.6] | 19.6 [14.0-25.7] | 35.8 [29.7-42.1] | 9700 [7700-11000] | 36000 [34000-39000] | 66000 [62000-70000] | 0.20 [0.13-0.29] |
| Uganda | 35 [30-40] | 3.5 [2.9-4.1] | 13.7 [9.3-18.8] | 30.9 [25.2-36.6] | 98000 [88000-110000] | 490000 [460000-550000] | 820000 [760000-910000] | 0.20 [0.15-0.25] |
| United Republic of Tanzania | 35 [30-40] | 3.5 [3.2-3.9] | 19.6 [14.2-25.8] | 35.5 [29.8-41.2] | 110000 [93000-130000] | 610000 [570000-680000] | 1000000 [960000-1100000] | 0.08 [0.05-0.12] |
| Zambia | 35 [30-40] | 4.2 [3.7-4.9] | 19.0 [13.4-25.7] | 36.3 [30.4-42.7] | 82000 [73000-93000] | 520000 [490000-580000] | 850000 [790000-940000] | 0.15 [0.10-0.22] |
| Zimbabwe | 35 [30-40] | 2.9 [2.4-3.5] | 22.2 [16.2-28.7] | 52.8 [46.1-59.6] | 79000 [66000-92000] | 470000 [430000-520000] | 720000 [660000-790000] | 0.04 [0.03-0.05] |
