# Supplementary material for: Characterisation of populations at risk of sub-optimal dosing of artemisinin-based combination therapy in Africa
Source: PLOS Glob Public Health. 2023 Dec 1;3(12):e0002059. doi: 10.1371/journal.pgph.0002059 (PMC10691722; doi:10.1371/journal.pgph.0002059)
Supplement: S1 Table — (DOCX) [file pgph.0002059.s005.docx]

**S1 Table. Estimated number of uncomplicated *Pf* malaria cases (in million) in population at increased risk of sub-optimal dosing, by country**

|  | **Under-5 years old** | | | | | **5 to 14 years old** | | | | **Adults 15 years old and older** | | | | | |
| --- | --- | --- | --- | --- | --- | --- | --- | --- | --- | --- | --- | --- | --- | --- | --- |
| **Country** | **Uncomplicated malaria** | **No risk** | **Hyper** | **HIV** | **wasted** | **Uncomplicated malaria** | **No risk** | **Hyper** | **HIV** | **Uncomplicated malaria** | **No risk** | **Hyper** | **HIV** | **Overweight** | **Pregnant** |
| Angola | 1.519637 | 1.265279 | 0.1470282 | 0.0029837 | 0.1043462 | 2.461651 | 2.206127 | 0.2504419 | 0.0050823 | 2.519059 | 1.59541 | 0.165329 | 0.0320354 | 0.3931147 | 0.3331702 |
| Benin | 0.8471678 | 0.7219456 | 0.0819553 | 0.0009085 | 0.0423584 | 0.9481438 | 0.8505223 | 0.0965513 | 0.0010703 | 0.6926551 | 0.4353862 | 0.0449636 | 0.0043233 | 0.1392336 | 0.0687483 |
| Burkina Faso | 3.546081 | 2.924974 | 0.3318465 | 0.0035361 | 0.2857248 | 4.009134 | 3.596536 | 0.4082476 | 0.0043502 | 2.95318 | 2.06203 | 0.209651 | 0.0129744 | 0.3628515 | 0.3056727 |
| Burundi | 1.328272 | 1.127423 | 0.1287614 | 0.0015633 | 0.0705243 | 1.798818 | 1.613426 | 0.1831677 | 0.0022239 | 1.551768 | 1.037049 | 0.1101438 | 0.0126605 | 0.2357672 | 0.1561475 |
| Cameroon | 0.637026 | 0.5276369 | 0.0619899 | 0.0013581 | 0.0460412 | 1.065231 | 0.9545411 | 0.1083167 | 0.002373 | 1.124331 | 0.6379603 | 0.0676348 | 0.0244555 | 0.2673046 | 0.1269761 |
| Central African Republic | 0.5416424 | 0.4526885 | 0.0522567 | 0.0010001 | 0.0356971 | 0.6650491 | 0.5960715 | 0.0676823 | 0.0012953 | 0.5253075 | 0.3304356 | 0.0343696 | 0.013464 | 0.0865712 | 0.0604671 |
| Chad | 0.2488755 | 0.1927544 | 0.0218265 | 0.0002724 | 0.0340221 | 0.5355859 | 0.4803507 | 0.0545543 | 0.000681 | 0.7353981 | 0.4784229 | 0.0505755 | 0.0078668 | 0.1090694 | 0.0894636 |
| Congo | 0.0187532 | 0.01534 | 0.0017478 | 0.0000637 | 0.0016017 | 0.032654 | 0.0292178 | 0.0033153 | 0.0001208 | 0.0358599 | 0.0229093 | 0.0022663 | 0.0008877 | 0.0062376 | 0.0035591 |
| Côte d'Ivoire | 1.351585 | 1.121175 | 0.1292113 | 0.001583 | 0.0996158 | 1.89951 | 1.703751 | 0.1933897 | 0.0023692 | 1.696275 | 1.041463 | 0.1055528 | 0.0263836 | 0.338071 | 0.1848043 |
| Democratic Republic of the Congo | 7.492901 | 6.135825 | 0.7141858 | 0.009868 | 0.6330234 | 8.463777 | 7.589983 | 0.8618853 | 0.0119088 | 6.229954 | 4.107325 | 0.4204895 | 0.0461308 | 0.8820429 | 0.7739654 |
| Djibouti | 0.0063692 | 0.0039751 | 0.00051 | 0.000000 | 0.0018842 | 0.0208248 | 0.0187007 | 0.0021241 | 0.000000 | 0.0575662 | 0.0391007 | 0.0033863 | 0.0002389 | 0.0106179 | 0.0042224 |
| Equatorial Guinea | 0.006089 | 0.0051611 | 0.0005984 | 0.0000319 | 0.0002975 | 0.0096605 | 0.0086284 | 0.0009798 | 0.0000522 | 0.0096883 | 0.0061798 | 0.0006339 | 0.0006087 | 0.0013724 | 0.0008935 |
| Eritrea | 0.0055831 | 0.0041437 | 0.0004863 | 0.000000 | 0.0009531 | 0.0182269 | 0.0163677 | 0.0018591 | 0.000000 | 0.0501172 | 0.0352148 | 0.0036878 | 0.000183 | 0.0066534 | 0.0043781 |
| Ethiopia | 0.1420486 | 0.1176041 | 0.0134332 | 0.0000963 | 0.010915 | 0.4513118 | 0.4049914 | 0.0459909 | 0.0003295 | 1.147699 | 0.8447541 | 0.0845294 | 0.0074609 | 0.1107979 | 0.1001562 |
| Gabon | 0.0110179 | 0.009387 | 0.0010819 | 0.0000276 | 0.0005214 | 0.0190624 | 0.0170752 | 0.0019376 | 0.0000495 | 0.0208024 | 0.0104736 | 0.0011615 | 0.000461 | 0.0066696 | 0.0020367 |
| Gambia | 0.0068233 | 0.0057989 | 0.0006592 | 0.0000079 | 0.0003573 | 0.0205372 | 0.0184215 | 0.0020907 | 0.0000251 | 0.0459231 | 0.0276262 | 0.0028712 | 0.0004889 | 0.0098618 | 0.005075 |
| Ghana | 1.022737 | 0.8424542 | 0.0969923 | 0.0021046 | 0.081186 | 1.977506 | 1.771918 | 0.2012219 | 0.0043663 | 2.412983 | 1.365574 | 0.1544563 | 0.0353966 | 0.6553231 | 0.2022331 |
| Guinea | 0.5623924 | 0.4487304 | 0.0519957 | 0.0005875 | 0.0610788 | 0.766376 | 0.6874603 | 0.078034 | 0.0008817 | 0.6649424 | 0.4369184 | 0.0447946 | 0.0059662 | 0.1110227 | 0.0662405 |
| Guinea-Bissau | 0.0159954 | 0.0135469 | 0.0015418 | 0.0000453 | 0.0008615 | 0.0459234 | 0.041122 | 0.0046643 | 0.000137 | 0.0943215 | 0.0591311 | 0.0060462 | 0.0018985 | 0.0184977 | 0.008748 |
| Kenya | 0.3228752 | 0.2760626 | 0.0314224 | 0.0010252 | 0.0143651 | 0.9941065 | 0.8898233 | 0.1009885 | 0.0032948 | 2.335376 | 1.458213 | 0.1586571 | 0.0813788 | 0.4580819 | 0.1790449 |
| Liberia | 0.3340379 | 0.2889855 | 0.0328697 | 0.0002866 | 0.0118961 | 0.3288897 | 0.2950954 | 0.0335022 | 0.0002921 | 0.2174621 | 0.1281038 | 0.0139733 | 0.001563 | 0.0522366 | 0.0215853 |
| Madagascar | 0.2081906 | 0.1728921 | 0.0198727 | 0.0000245 | 0.0154014 | 0.5831084 | 0.5235691 | 0.0594659 | 0.0000733 | 1.151718 | 0.7922181 | 0.0824729 | 0.001818 | 0.1701874 | 0.1050221 |
| Malawi | 1.382397 | 1.227523 | 0.1391329 | 0.0082326 | 0.0075092 | 2.602144 | 2.323077 | 0.2634766 | 0.0155901 | 3.088796 | 1.8341005 | 0.202462 | 0.2157045 | 0.5454326 | 0.2910964 |
| Mali | 0.5618994 | 0.453863 | 0.0519115 | 0.0005853 | 0.0555395 | 0.9826014 | 0.8813863 | 0.1000865 | 0.0011285 | 1.083624 | 0.6735814 | 0.0705801 | 0.0077549 | 0.1946788 | 0.1370283 |
| Mauritania | 0.0012479 | 0.0009816 | 0.0001127 | 0.000000 | 0.0001537 | 0.0035817 | 0.0032164 | 0.0003653 | 0.000000 | 0.0073524 | 0.0045243 | 0.0004481 | 0.0000147 | 0.0015888 | 0.0007765 |
| Mozambique | 3.124037 | 2.643346 | 0.3017353 | 0.0193018 | 0.1596539 | 4.329696 | 3.864283 | 0.437431 | 0.0279821 | 3.816227 | 2.331464 | 0.2349083 | 0.2831425 | 0.5441681 | 0.4225443 |
| Namibia | 0.0009547 | 0.000773 | 0.0000896 | 0.00000738 | 0.0000847 | 0.0031427 | 0.002799 | 0.0003176 | 0.0000262 | 0.0089375 | 0.0044965 | 0.0004537 | 0.0010104 | 0.0022564 | 0.0007205 |
| Niger | 1.019841 | 0.8253734 | 0.0938043 | 0.0001803 | 0.1004834 | 1.642589 | 1.474768 | 0.1674991 | 0.0003219 | 1.671562 | 1.073494 | 0.1164968 | 0.0028467 | 0.2645776 | 0.2141469 |
| Nigeria | 4.996346 | 4.179758 | 0.4758179 | 0.0044866 | 0.3362828 | 7.001035 | 6.28123 | 0.7130812 | 0.0067238 | 6.234547 | 3.926473 | 0.40502 | 0.0570973 | 1.178147 | 0.6678104 |
| Rwanda | 0.1732536 | 0.1536277 | 0.0174369 | 0.0002832 | 0.0019058 | 0.5417392 | 0.4857148 | 0.0551291 | 0.0008953 | 1.321444 | 0.8385353 | 0.0911329 | 0.0247724 | 0.2622758 | 0.1047279 |
| Sao Tome and Principe | 0.0001451 | 0.0001215 | 0.0000142 | 0.000000 | 0.00000947 | 0.0004755 | 0.000427 | 0.0000485 | 0.000000 | 0.0013234 | 0.0008651 | 0.0000815 | 0.000000 | 0.0002654 | 0.0001113 |
| Senegal | 0.0421516 | 0.0338376 | 0.003949 | 0.0000219 | 0.0043432 | 0.1250367 | 0.1122196 | 0.0127465 | 0.0000705 | 0.2717966 | 0.1763353 | 0.0182208 | 0.000999 | 0.049162 | 0.0270795 |
| Sierra Leone | 0.2148054 | 0.1817415 | 0.0206574 | 0.0005389 | 0.0118676 | 0.2585614 | 0.2315909 | 0.0262847 | 0.0006857 | 0.2007528 | 0.1266908 | 0.0134998 | 0.0021746 | 0.0395661 | 0.0188214 |
| Somalia | 0.0030478 | 0.0022614 | 0.0002664 | 0.00000021 | 0.0005198 | 0.0083042 | 0.0074566 | 0.0008469 | 0.00000067 | 0.015733 | 0.0100501 | 0.0010176 | 0.00000877 | 0.0024976 | 0.0021589 |
| South Sudan | 0.0557908 | 0.0376647 | 0.0043843 | 0.0001138 | 0.013628 | 0.0795812 | 0.0712809 | 0.0080904 | 0.00021 | 0.0720687 | 0.0450723 | 0.0047327 | 0.0016173 | 0.0145562 | 0.0060903 |
| Sudan | 0.162313 | 0.122439 | 0.0138542 | 0.000021 | 0.0259988 | 0.4811697 | 0.4320269 | 0.0490683 | 0.0000745 | 1.044691 | 0.6843156 | 0.0696756 | 0.0012146 | 0.1852993 | 0.1041858 |
| Togo | 0.4868022 | 0.4136258 | 0.0466907 | 0.0009259 | 0.0255598 | 0.8853036 | 0.7934732 | 0.0900448 | 0.0017856 | 1.015015 | 0.6348781 | 0.0676576 | 0.0146519 | 0.202273 | 0.0955541 |
| Uganda | 2.882805 | 2.478802 | 0.2823833 | 0.0115672 | 0.1100523 | 5.233057 | 4.680106 | 0.5311924 | 0.0217591 | 5.988752 | 3.658216 | 0.4052573 | 0.2798076 | 1.000797 | 0.6446743 |
| United Republic of Tanzania | 0.7093612 | 0.608555 | 0.0695387 | 0.002025 | 0.0292425 | 1.859966 | 1.665518 | 0.1889457 | 0.0055023 | 3.330592 | 1.977723 | 0.2128912 | 0.112744 | 0.6618054 | 0.3654288 |
| Zambia | 1.351486 | 1.15546 | 0.130752 | 0.008511 | 0.0567624 | 2.861937 | 2.554101 | 0.2890222 | 0.0188133 | 3.85636 | 2.249754 | 0.2285184 | 0.3284861 | 0.6172423 | 0.4323591 |
| Zimbabwe | 0.0415801 | 0.0357376 | 0.0040677 | 0.0004632 | 0.0013115 | 0.1246415 | 0.1106538 | 0.0125577 | 0.00143 | 0.2764426 | 0.1268064 | 0.0140916 | 0.0334147 | 0.0768158 | 0.025314 |
