# Supplementary material for: Characterisation of populations at risk of sub-optimal dosing of artemisinin-based combination therapy in Africa
Source: PLOS Glob Public Health. 2023 Dec 1;3(12):e0002059. doi: 10.1371/journal.pgph.0002059 (PMC10691722; doi:10.1371/journal.pgph.0002059)
Supplement: S2 Table — (DOCX) [file pgph.0002059.s006.docx]

**S2 Table. Uncertainty limits of the estimated number of the uncomplicated *Pf* malaria cases (in million) in population at increased risk of sub-optimal dosing by country**

| **Country** | **Wasted**  **(in <5 years)** | **Pregnancy**  **(in females >14 years)** | **Overweight**  **(in >14 years)** | **PLHIV**  **(in all ages)** | **Hyperparasitaemia (in all ages)** |
| --- | --- | --- | --- | --- | --- |
| Angola | 0.0916-0.1192 | 0.3163-0.3546 | 0.2877-0.5059 | 0.0337-0.0499 | 0.5595-0.7323 |
| Benin | 0.0382-0.0457 | 0.0664-0.0711 | 0.1122-0.1682 | 0.0054-0.0080 | 0.2189-0.2960 |
| Burkina Faso | 0.2681-0.2998 | 0.2950-0.3164 | 0.2789-0.4523 | 0.0168-0.0252 | 0.9295-1.2602 |
| Burundi | 0.0632-0.0764 | 0.1523-0.1560 | 0.1773-0.3005 | 0.0141-0.0191 | 0.4143-0.5578 |
| Cameroon | 0.0364-0.0578 | 0.1197-0.1362 | 0.2208-0.3159 | 0.0257-0.0309 | 0.2356-0.3113 |
| Central African Republic | 0.0323-0.0398 | 0.0578-0.0639 | 0.0643-0.1113 | 0.0130-0.0195 | 0.1520-0.2028 |
| Chad | 0.0323-0.0360 | 0.0855-0.0945 | 0.0836-0.1369 | 0.0071-0.0107 | 0.1260-0.1654 |
| Congo | 0.0014-0.0018 | 0.0033-0.0038 | 0.0048-0.0077 | 0.0007-0.0016 | 0.0073-0.0095 |
| Côte d'Ivoire | 0.0898-0.1094 | 0.1778-0.1918 | 0.2730-0.4058 | 0.0265-0.0351 | 0.4222-0.5633 |
| Democratic Republic of the Congo | 0.5638-0.7220 | 0.7385-0.8191 | 0.6510-1.1321 | 0.0580-0.0796 | 1.9628-2.6310 |
| Djibouti | 0.0017-0.0021 | 0.0040-0.0044 | 0.0084-0.0129 | 0.0002-0.0003 | 0.0062-0.0075 |
| Equatorial Guinea | 0.0003-0.0003 | 0.0008-0.0010 | 0.0010-0.0017 | 0.0005-0.0010 | 0.0022-0.0029 |
| Eritrea | 0.0009-0.0010 | 0.0042-0.0045 | 0.0050-0.0085 | 0.0001-0.0002 | 0.0061-0.0077 |
| Ethiopia | 0.0109-0.0109 | 0.0974-0.1029 | 0.0831-0.1412 | 0.0063-0.0096 | 0.1440-0.1860 |
| Gabon | 0.0004-0.0007 | 0.0019-0.0022 | 0.0052-0.0081 | 0.0004-0.0007 | 0.0042-0.0054 |
| Gambia | 0.0004-0.0004 | 0.0049-0.0052 | 0.0080-0.0119 | 0.0004-0.0007 | 0.0057-0.0072 |
| Ghana | 0.0728-0.0907 | 0.1930-0.2115 | 0.5444-0.7728 | 0.0360-0.0487 | 0.4485-0.5921 |
| Guinea | 0.0538-0.0697 | 0.0638-0.0687 | 0.0880-0.1365 | 0.0068-0.0081 | 0.1721-0.2301 |
| Guinea-Bissau | 0.0008-0.0010 | 0.0084-0.0091 | 0.0146-0.0227 | 0.0018-0.0023 | 0.0124-0.0157 |
| Kenya | 0.0130-0.0157 | 0.1732-0.1849 | 0.3625-0.5601 | 0.0741-0.0999 | 0.2922-0.3748 |
| Liberia | 0.0119-0.0119 | 0.0207-0.0224 | 0.0421-0.0627 | 0.0017-0.0027 | 0.0785-0.1068 |
| Madagascar | 0.0142-0.0168 | 0.1020-0.1080 | 0.1303-0.2155 | 0.0015-0.0025 | 0.1616-0.2091 |
| Malawi | 0.0050-0.0125 | 0.2830-0.2992 | 0.4250-0.6757 | 0.2218-0.2615 | 0.5970-0.7943 |
| Mali | 0.0514-0.0603 | 0.1330-0.1410 | 0.1543-0.2388 | 0.0077-0.0116 | 0.2201-0.2915 |
| Mauritania | 0.0001-0.0002 | 0.0007-0.0008 | 0.0013-0.0019 | 0.0000-0.0000 | 0.0009-0.0012 |
| Mozambique | 0.1379-0.1851 | 0.4121-0.4329 | 0.4402-0.6504 | 0.2677-0.4145 | 0.9607-1.2789 |
| Namibia | 0.0001-0.0001 | 0.0006-0.0009 | 0.0019-0.0026 | 0.0010-0.0011 | 0.0009-0.0011 |
| Niger | 0.0882-0.1138 | 0.2086-0.2197 | 0.2019-0.3364 | 0.0029-0.0037 | 0.3723-0.4964 |
| Nigeria | 0.3104-0.3622 | 0.6455-0.6901 | 0.9685-1.3952 | 0.0503-0.0930 | 1.5660-2.1054 |
| Rwanda | 0.0019-0.0019 | 0.1015-0.1079 | 0.2063-0.3207 | 0.0238-0.0299 | 0.1640-0.2114 |
| Sao Tome and Principe | 0.0000-0.0000 | 0.0001-0.0001 | 0.0002-0.0003 | 0.0000-0.0000 | 0.0001-0.0002 |
| Senegal | 0.0040-0.0048 | 0.0260-0.0281 | 0.0392-0.0598 | 0.0010-0.0013 | 0.0350-0.0450 |
| Sierra Leone | 0.0103-0.0136 | 0.0181-0.0196 | 0.0315-0.0480 | 0.0029-0.0040 | 0.0593-0.0799 |
| Somalia | 0.0004-0.0006 | 0.0021-0.0022 | 0.0019-0.0031 | 0.0000-0.0000 | 0.0021-0.0028 |
| South Sudan | 0.0127-0.0147 | 0.0059-0.0063 | 0.0115-0.0179 | 0.0015-0.0025 | 0.0171-0.0225 |
| Sudan | 0.0242-0.0278 | 0.0949-0.1176 | 0.1462-0.2273 | 0.0011-0.0016 | 0.1339-0.1691 |
| Togo | 0.0224-0.0296 | 0.0918-0.0993 | 0.1603-0.2464 | 0.0159-0.019 | 0.2023-0.2677 |
| Uganda | 0.0912-0.1289 | 0.6289-0.6605 | 0.7809-1.2295 | 0.2906-0.3492 | 1.2006-1.6045 |
| United Republic of Tanzania | 0.0267-0.0326 | 0.3564-0.3745 | 0.5323-0.7969 | 0.1135-0.1335 | 0.4697-0.6121 |
| Zambia | 0.0500-0.0662 | 0.4209-0.4438 | 0.4959-0.7479 | 0.3313-0.3954 | 0.6430-0.8444 |
| Zimbabwe | 0.0011-0.0016 | 0.0245-0.0261 | 0.0652-0.0883 | 0.0322-0.0390 | 0.0311-0.0393 |
