# Supplementary material for: Characterisation of populations at risk of sub-optimal dosing of artemisinin-based combination therapy in Africa
Source: PLOS Glob Public Health. 2023 Dec 1;3(12):e0002059. doi: 10.1371/journal.pgph.0002059 (PMC10691722; doi:10.1371/journal.pgph.0002059)
Supplement: S3 Table — (DOCX) [file pgph.0002059.s007.docx]

## **S3 Table. Uncertainty limits of the estimated number of the uncomplicated *Pf* malaria cases (in million) in population at increased risk of sub-optimal dosing, by region**

|  |  | **Wasted (in <5 years)** | **Pregnancy (in females >14 years)** | **Overweight (in >14 years)** | **PLHIV (in all ages)** | **Hyperparasitaemia (in all ages)** |
| --- | --- | --- | --- | --- | --- | --- |
| Total estimated cases | N | 2.23 - 2.79 | 6.13 - 6.61 | 8.12 - 12.58 | 1.70 - 2.21 | 13.03 - 17.34 |
| (41 countries) | % | 1.5 - 1.8 | 4.0 - 4.3 | 5.3 - 8.2 | 1.1 - 1.4 | 8.5 - 11.3 |
| *By region* |  |  |  |  |  |  |
| Northern Africa | N | 0.02 - 0.03 | 0.09 - 0.12 | 0.15 - 0.23 | 0.001 - 0.002 | 0.13 - 0.17 |
| (1 country) | % | 0.0 - 0.0 | 0.0 - 0.1 | 0.0 - 0.1 | 0.0 - 0.0 | 0.0 - 0.1 |
| East Africa | N | 0.43 - 0.57 | 2.77 - 2.92 | 3.73 - 5.77 | 1.38 - 1.76 | 5.11 - 6.75 |
| (15 countries) | % | 0.2 - 0.4 | 1.8 - 1.9 | 2.4 - 3.7 | 0.9 - 1.1 | 3.3 - 4.4 |
| West Africa | N | 1.02 - 1.21 | 1.95 - 2.09 | 2.92 - 4.36 | 0.18 - 0.26 | 4.74 - 6.36 |
| (15 countries) | % | 0.6 - 0.8 | 1.2 - 1.4 | 1.9 - 2.8 | 0.1 - 0.2 | 3.0 - 4.2 |
| Central Africa | N | 0.76 - 0.98 | 1.32 - 1.48 | 1.32 - 2.22 | 0.14 - 0.19 | 3.05 - 4.06 |
| (9 countries) | % | 0.5 - 0.6 | 0.8 - 1.0 | 0.9 - 1.5 | 0.1 - 0.1 | 1.9 - 2.7 |
| Southern Africa | N | 0.00007 - 0.0001 | 0.0006 - 0.0009 | 0.001 - 0.003 | 0.001 - 0.001 | 0.0008 - 0.001 |
| (1 country) | % | 0.0 - 0.0 | 0.0 - 0.1 | 0.0 - 0.1 | 0.0 - 0.0 | 0.0 - 0.1 |
| *By endemicity^1^* |  |  |  |  |  |  |
| Hypo-endemic | N | 0.10 - 0.12 | 1.00 - 1.08 | 1.62 - 2.48 | 0.26 - 0.32 | 1.46 - 1.89 |
| (16 countries) | % | 0.0 - 0.1 | 0.6 - 0.7 | 1.0 - 1.6 | 0.1 - 0.2 | 0.9 - 1.2 |
| Meso-endemic | N | 2.13 - 2.67 | 5.14 - 5.53 | 6.50 - 10.10 | 1.44 - 1.89 | 11.57 - 15.45 |
| (25 countries) | % | 1.4 - 1.7 | 3.4 - 3.6 | 4.2 - 6.6 | 0.9 - 1.2 | 7.6 - 10.1 |

The lowest and highest estimates were based on range values as reported in S4_Text, Table D. Percentages are in total of estimated uncomplicated cases. The list of countries by region and by endemicity areas are reported in S4_Text, Table A.

^1^ Hypo-endemicity: *Plasmodium falciparum (Pf)* prevalence in 2-9 years old <10%; Meso-endemicity: *Pf* prevalence in 2-9 years old 11-50%. No country was reported as hyper-endemic in 2020.

In <5 years: children under 5 years old; in >14 years: adults aged 15 years and older.
