# Supplementary material for: Characterisation of populations at risk of sub-optimal dosing of artemisinin-based combination therapy in Africa
Source: PLOS Glob Public Health. 2023 Dec 1;3(12):e0002059. doi: 10.1371/journal.pgph.0002059 (PMC10691722; doi:10.1371/journal.pgph.0002059)
Supplement: S4 Table — (DOCX) [file pgph.0002059.s008.docx]

**S4 Table. Estimates of risk of malaria recrudescence in populations of interest, extracted from literature**

Highlighted rows correspond to the relevant comparisons and these estimates were used to calculate pooled estimates reported in the main text and in Table 2.

| **PUBMED ID** | **Group /Variable** | **ACT** | **Reference Group** | **Day** | **HR** | **95% CI** | **p-value** | **Author** | **Year** | **Reference** | **Information retrieved in :** |
| --- | --- | --- | --- | --- | --- | --- | --- | --- | --- | --- | --- |
| **Children<5 years of age with malnutrition** | | | | | | | | | | | |
| 25788162 | 1-<3 years underweight (waz<-2) | AL-Africa | 12+ years | 28 | 4.05 | 1.78–9.18 | 0.001 | WWARN A-L dose impact Study Group | 2015 | AL Dose Impact Study | table5 |
| 25788162 | 1-<3 years underweight (waz<-2) | AL-Asia | 12+ years | 28 | 3.97 | 0.87-18.16 | 0.075 | WWARN A-L dose impact Study Group | 2015 | AL Dose Impact Study | table5 |
| 25788162 | 1-<3 years underweight (waz<-2) | AL | 1-<3 years not underweight | 28 | 1.56 | 1.04-2.43 | 0.033 | WWARN A-L dose impact Study Group | 2015 | AL Dose Impact Study | text-p6; HR not reported as adjusted HR |
| 25788162 | 1-<3 years underweight (waz<-2) | AL-Africa | 1-<3 years not underweight | 28 | 1.66 | 1.05–2.63 | 0.028 | WWARN A-L dose impact Study Group | 2015 | AL Dose Impact Study | text-p6 |
| 25788162 | 1-<3 years underweight (waz<-2) | AL-Asia | 1-<3 years, not underweight | 28 | 1.07 | 0.17-6.78 | 0.94 | WWARN A-L dose impact Study Group | 2015 | AL Dose Impact Study | text-p6 |
|  | 1-<5 years, wasted (whz<-2) | AL, AS-AQ, DP, AMSQ | 1-<5 years, not wasted | 42 | 1.41 | 1.07-1.86 | 0.013 | WWARN Malnutrition Study Group | 2016 | Malnutrition Study | Data presented at the 65^th^ ASTMH annual meeting in 2016 |
| **Baseline Parasitaemia / Hyperparasitaemia** | | | | | | | | | | | |
| 26381375 | Parasitaemia 10-fold | AL |  | 42 | 1.87 | 1.22-2.87 | 0.004 | WWARN PK-PD Study Group | 2015 | AL PK/PD Study | table3 |
| 25888957 | Parasitaemia 10-fold | AS-AQ |  | 28 | 1.39 | 1.1-1.74 | 0.005 | WWARN AS-AQ Study Group | 2015 | AS-AQ Study | text-p8 |
| 25888957 | Parasitaemia>100000 parasites per μL | AS-AQ | Parasitemia <=100000 | 28 | 1.41 | 0.98-2.05 | 0.066 | WWARN AS-AQ Study Group | 2015 | AS-AQ Study | table6, HR reported in univariable analysis only |
| 25888957 | Parasitaemia>100000 parasites per μL | AS-AQ | Parasitemia <=100000 | 28 | 1.36 | 0.95-1.97 |  | WWARN AS-AQ Study Group | 2015 | AS-AQ Study | not in the published paper - personal communication from authors |
| 25788162 | Parasitaemia 10-fold | AL |  | 28 | 1.41 | 1.15–1.74 | 0.0012 | WWARN A-L dose impact Study Group | 2015 | AL Dose Impact Study | table4 |
| 25788162 | Parasitaemia>100000 parasites per μL | AL-Africa | Parasitemia <=100000 | 28 | 1.44 | 0.99–2.1 | 0.054 | WWARN A-L dose impact Study Group | 2015 | AL Dose Impact Study | table5 |
| 25788162 | Parasitaemia>100000 parasites per μL | AL-Asia | Parasitemia <=100000 | 28 | 1.76 | 0.78-3.97 | 0.18 | WWARN A-L dose impact Study Group | 2015 | AL Dose Impact Study | table5 |
| 24311989 | Parasitaemia 10-fold | DP |  | 42 | 1.23 | 1.08-1.41 | 0.003 | WWARN DP Study Group | 2013 | DP Study | table6 |
| 24311989 | Parasitaemia>100000 parasites per μL | DP | Parasitemia <=100000 | 42 | 1.72 | 1.07-2.77 |  | WWARN DP Study Group | 2013 | DP Study | not in the published paper - personal communication from authors |
| 32530424 | Parasitaemia 10-fold in pregnancy | AL, AAP, AC, AS, ASAQ, ASMQ, ASSP, DP, Q, QC |  | 28 | 1.93 | 1.61-2.32 | <0.0001 | Saito, M | 2020 | ACTs in pregnancy | text-p946 + table3 |
| 32530424 | Parasitaemia>100000 parasites per μLin pregnancy | AL, AAP, AC, AS, ASAQ, ASMQ, ASSP, DP, Q, QC | Parasitemia <=100000 | 28 | 1.74 | 0.76-4.00 | 0.19 | Saito, M | 2020 | ACTs in pregnancy | table3; HR reported in univariable analysis only |
