# Supplementary material for: Characterisation of populations at risk of sub-optimal dosing of artemisinin-based combination therapy in Africa
Source: PLOS Glob Public Health. 2023 Dec 1;3(12):e0002059. doi: 10.1371/journal.pgph.0002059 (PMC10691722; doi:10.1371/journal.pgph.0002059)
Supplement: S5 Table — (DOCX) [file pgph.0002059.s009.docx]

## **S5 Table: Uncertainty limits of the estimated excess failures (in millions) in different risk groups.**

Different treatment failure rates and a range of assumed Hazard Ratios (HR) for people living with HIV, pregnant women, and overweight adults were considered.

|  | **Main analysis** | | | **Sensitivity analyses** | | | | | |
| --- | --- | --- | --- | --- | --- | --- | --- | --- | --- |
|  | **Assumed HR = 1.5 for  PLHIV, Overweight and Pregnant** | | | **Assumed HR = 1.2 for  PLHIV, Overweight and Pregnant** | | | **Assumed HR = 2 for  PLHIV, Overweight and Pregnant** | | |
| Risk groups | **2% failure** | **5% failure** | **8% failure** | **2% failure** | **5% failure** | **8% failure** | **2% failure** | **5% failure** | **8% failure** |
| **<5 years** |  |  |  |  |  |  |  |  |  |
| Hyperparasitaemic | 0.015 – 0.083 | 0.036 – 0.207 | 0.058 – 0.331 | 0.015 – 0.083 | 0.036 – 0.207 | 0.058 – 0.331 | 0.015 – 0.083 | 0.036 – 0.207 | 0.058 – 0.331 |
| PLHIV | 0.001 – 0.001 | 0.002 – 0.003 | 0.003 – 0.004 | 0.000 – 0.000 | 0.001 – 0.001 | 0.001 – 0.002 | 0.001 – 0.002 | 0.003 – 0.005 | 0.005 – 0.008 |
| Wasted | 0.003 – 0.048 | 0.008 – 0.120 | 0.013 – 0.192 | 0.003 – 0.048 | 0.008 – 0.120 | 0.013 – 0.192 | 0.003 – 0.048 | 0.008 – 0.120 | 0.013 – 0.192 |
| Sub-total for <5y | 0.018 – 0.132 | 0.046 – 0.329 | 0.073 – 0.526 | 0.018 – 0.131 | 0.045 – 0.327 | 0.072 – 0.524 | 0.019 – 0.133 | 0.048 – 0.332 | 0.076 – 0.530 |
| **5-14 years** |  |  |  |  |  |  |  |  |  |
| Hyperparasitaemic | 0.023 – 0.133 | 0.058 – 0.332 | 0.092 – 0.531 | 0.023 – 0.133 | 0.058 – 0.332 | 0.092 – 0.531 | 0.023 – 0.133 | 0.058 – 0.332 | 0.092 – 0.531 |
| PLHIV | 0.001 – 0.002 | 0.003 – 0.004 | 0.005 – 0.007 | 0.000 – 0.001 | 0.001 – 0.002 | 0.002 – 0.003 | 0.002 – 0.004 | 0.006 – 0.009 | 0.009 – 0.014 |
| Sub-total for 5-14y | 0.024 – 0.134 | 0.061 – 0.336 | 0.097 – 0.538 | 0.024 – 0.133 | 0.059 – 0.333 | 0.094 – 0.534 | 0.025 – 0.136 | 0.063 – 0.341 | 0.102 – 0.545 |
| **>14 years** |  |  |  |  |  |  |  |  |  |
| Hyperparasitaemic | 0.017 – 0.083 | 0.043 – 0.207 | 0.069 – 0.332 | 0.017 – 0.083 | 0.043 – 0.207 | 0.069 – 0.332 | 0.017 – 0.083 | 0.043 – 0.207 | 0.069 – 0.332 |
| PLHIV | 0.015 – 0.019 | 0.038 – 0.048 | 0.060 – 0.078 | 0.006 – 0.008 | 0.015 – 0.019 | 0.024 – 0.031 | 0.030 – 0.039 | 0.076 – 0.097 | 0.121 – 0.155 |
| Overweight | 0.081 – 0.126 | 0.203 – 0.314 | 0.324 – 0.503 | 0.032 – 0.050 | 0.081 – 0.126 | 0.130 – 0.201 | 0.162 – 0.252 | 0.406 – 0.629 | 0.649 – 1.006 |
| Pregnant | 0.061 – 0.066 | 0.154 – 0.165 | 0.246 – 0.264 | 0.025 – 0.026 | 0.061 – 0.066 | 0.098 – 0.106 | 0.123 – 0.132 | 0.307 – 0.330 | 0.491 – 0.529 |
| Sub-total for >14y | 0.175 – 0.294 | 0.437 – 0.736 | 0.699 – 1.177 | 0.080 – 0.167 | 0.201 – 0.419 | 0.321 – 0.670 | 0.332 – 0.505 | 0.831 – 1.264 | 1.330 – 2.022 |
| **Overall TOTAL failures** | 0.217 – 0.560 | 0.543 – 1.401 | 0.870 – 2.241 | 0.122 – 0.432 | 0.304 – 1.080 | 0.487 – 1.727 | 0.377 – 0.774 | 0.942 – 1.936 | 1.507 – 3.097 |
