# Supplementary figures and images for: Characterisation of populations at risk of sub-optimal dosing of artemisinin-based combination therapy in Africa
Source: PLOS Glob Public Health. 2023 Dec 1;3(12):e0002059. doi: 10.1371/journal.pgph.0002059 (PMC10691722; doi:10.1371/journal.pgph.0002059)

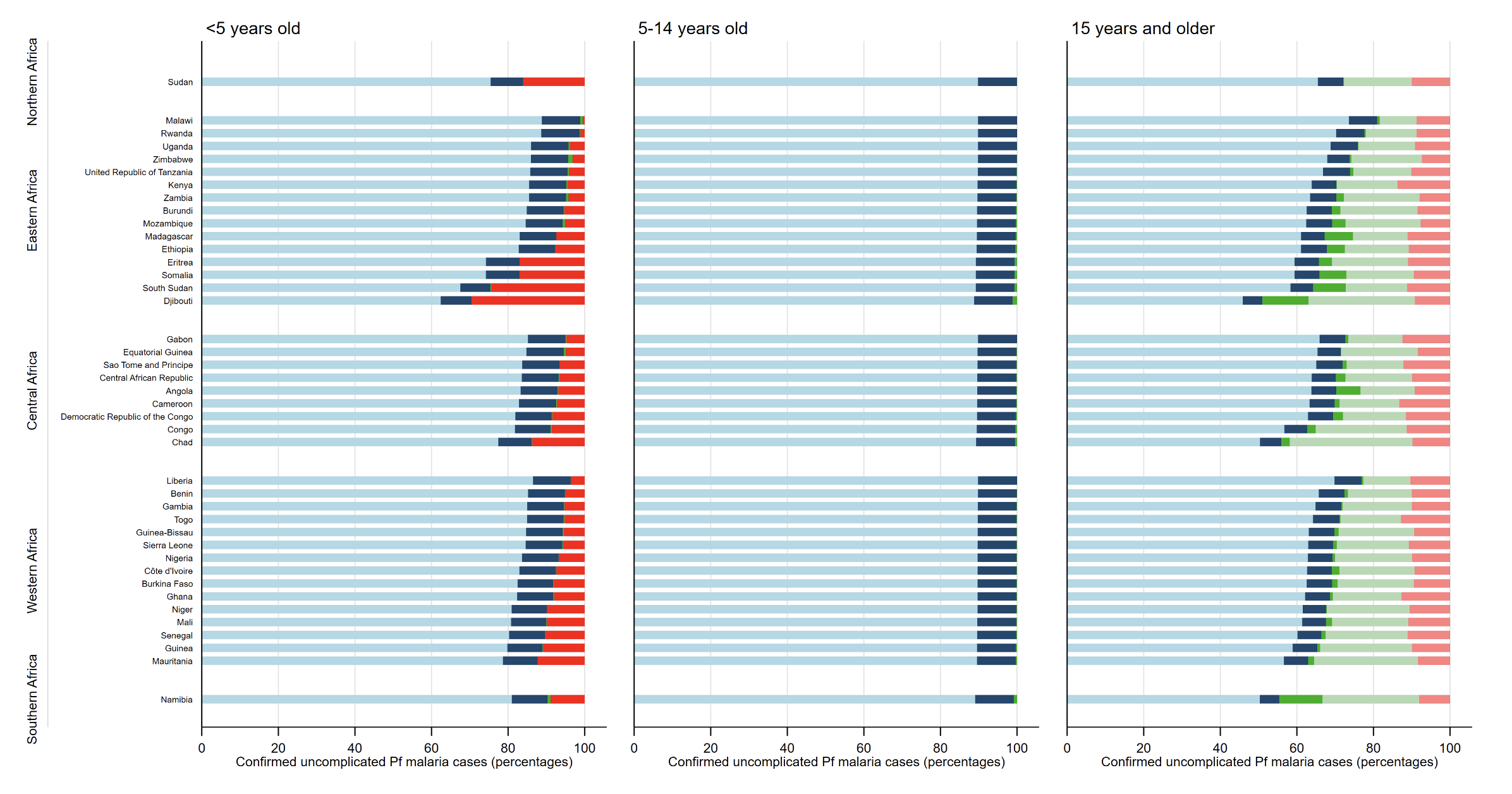

Supplement: S1 Fig — Data presented as percentage of total uncomplicated Pf malaria cases. Legend: pale blue = no risk factor; blue = patients with hyperparasitaemia; green = people living with HIV; red = children <5 years of age wasted; pale green = overweight adults; pale red = pregnant women. (TIFF) [file pgph.0002059.s010.tiff]

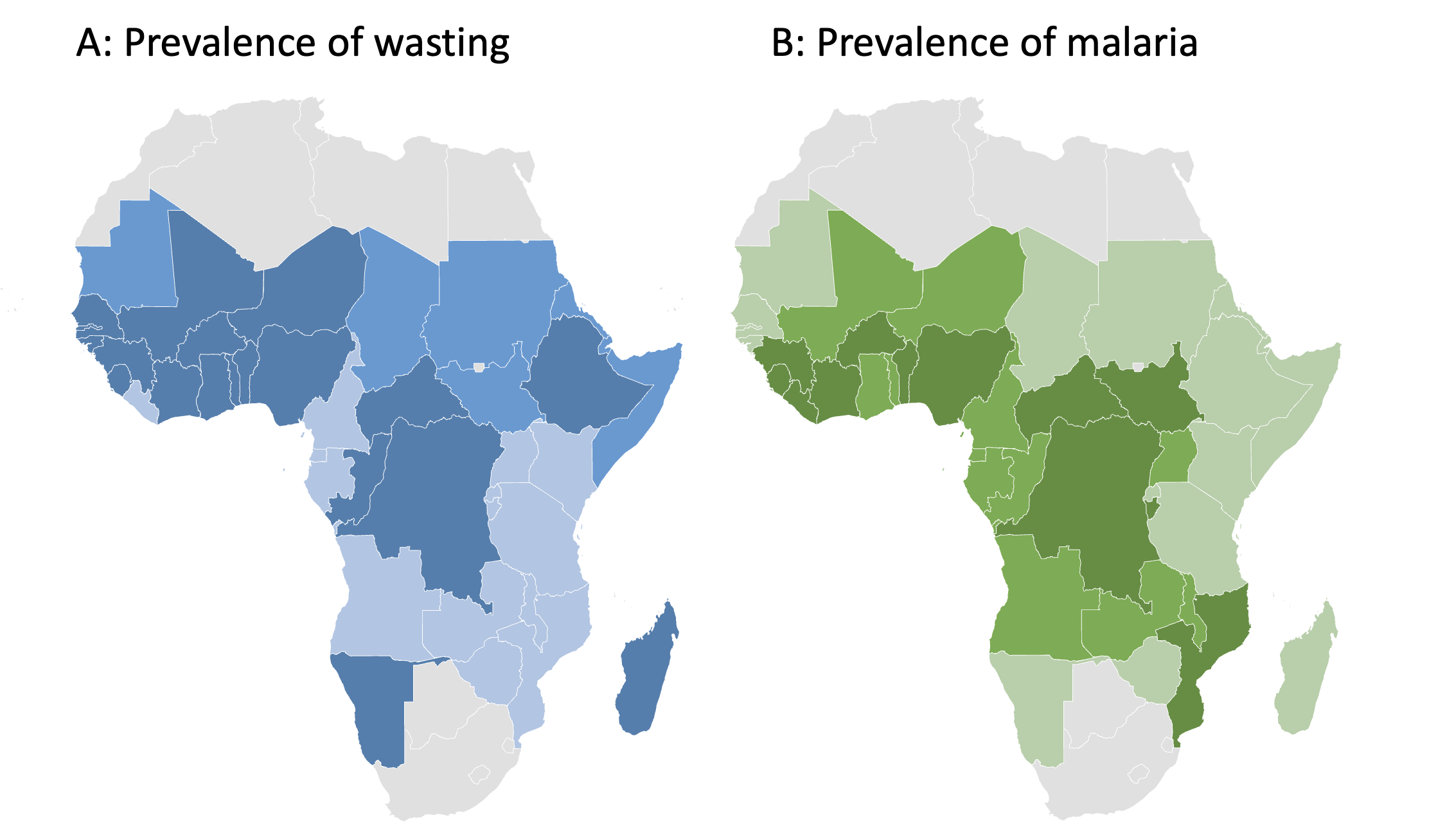

Supplement: S2 Fig — GeoNames, Microsoft, OpenStreetMap, TomTom. Panel A: moderate wasting (weight-for-height Z-score <-2) prevalence in children<5 years of age: <5% (pale blue), 5-<10% (blue) and ≥10% (dark blue). Latest data available from the WHO Global Health Observatory Data Repository [https://www.who.int/data/gho] are: 2020 for Rwanda, Nigeria, Gambia; 2019 for Guinea-Bissau, Ethiopia, Chad, Burundi, Liberia, Sierra Leone, Mali, Malawi, Senegal, CAR, Niger, Burkina Faso, Zimbabwe; 2018 for Guinea, Benin, Zambia, Cameroon, Tanzania, Mauritania, Madagascar; 2017 for Ghana, Togo, DRC; 2016 for Côte d’Ivoire, Uganda; 2015 for Mozambique, Angola; 2014 for Kenya, Congo, Sudan; 2013 for Namibia; 2012 for Gabon, Djibouti; 2011 for Equatorial Guinea; 2010 for South Sudan, Eritrea; 2009 for Somalia. Panel B: The 2020 Malaria Atlas Project [https://malariaatlas.org/] predicted age-standardized parasite rate for Pf malaria for children 2–10 years of age: ≤10% (pale green), 11–25% (green) and 26–50% (dark green). (TIFF) [file pgph.0002059.s011.tiff]

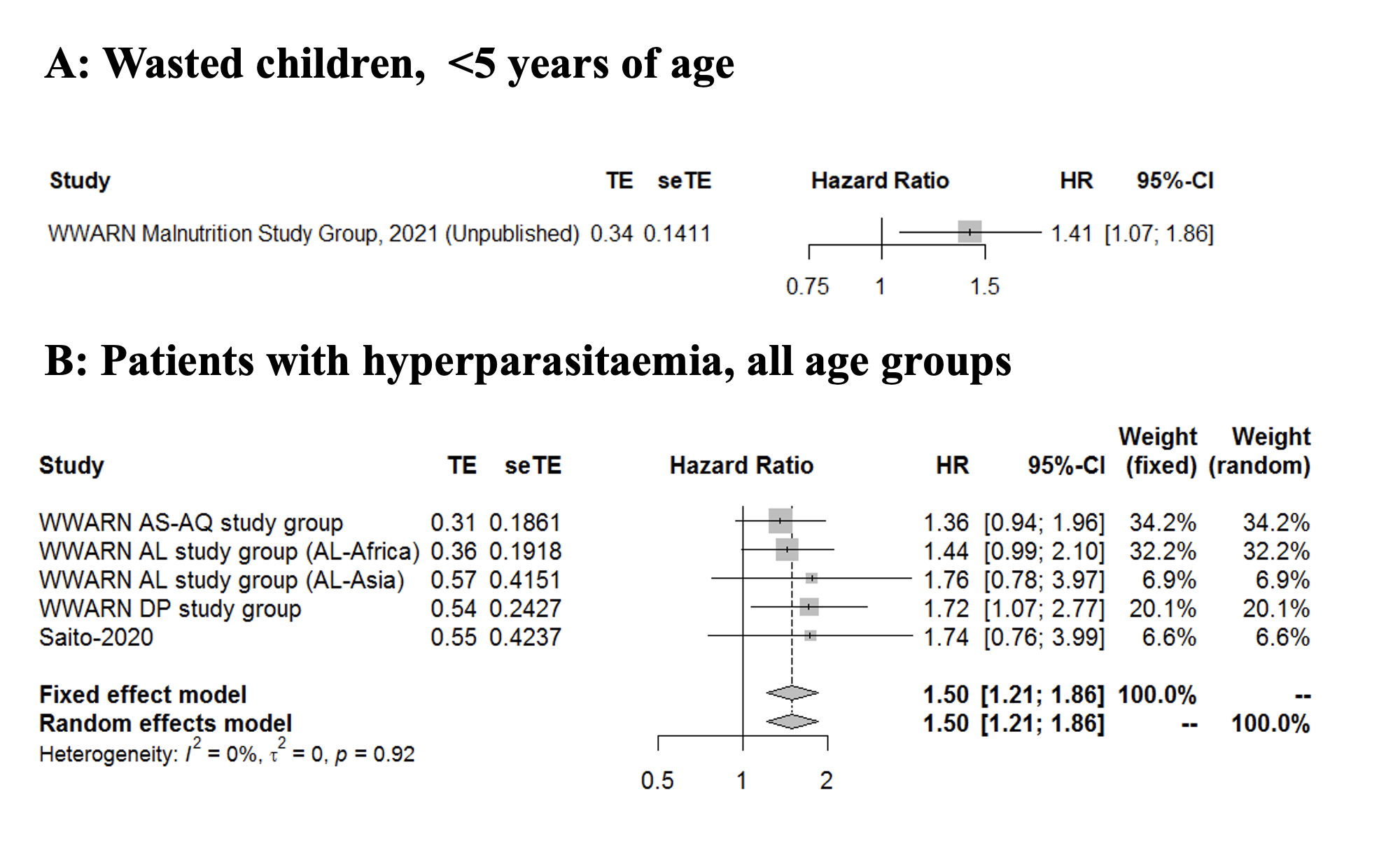

Supplement: S3 Fig — Panel B: For DP, the Hazard Ratio (HR) was reported for Day 42; for AL and AS-AQ, the HR was reported for Day 28. The assumption of proportional hazards was satisfied for all models. Comparator groups were children <5 years of age not wasted (Panel A); patients with parasitaemia <100000 parasites/microliter (Panel B). (TIFF) [file pgph.0002059.s012.tiff]
