# Supplementary material for: Characterisation of populations at risk of sub-optimal dosing of artemisinin-based combination therapy in Africa
Source: PLOS Glob Public Health. 2023 Dec 1;3(12):e0002059. doi: 10.1371/journal.pgph.0002059 (PMC10691722; doi:10.1371/journal.pgph.0002059)
Supplement: Attachment — Submitted filename: ResponseToReviewers_15OCT2023.docx [file pgph.0002059.s013.docx]

Dear Reviewers,

We very much appreciate your time and effort in reviewing this manuscript. Please find below our point-to-point responses.

Reviewer #1:

Underdosing of antimalarial drugs is clearly a serious problem now and in the future as an accelerant of antimalarial drug resistance. This study is important. It is well written and well explained. I have no major concerns, just some suggestions.

No response is required

General comments in order:

1. In the abstract, when referring to numbers of cases reported by WHO, please add the word “estimated” in case the reader actually believes the precision of these modelled values.

We added word `estimated’ in the abstract when referring to the number of cases and the excess number of treatment failures.

2. Line 58: “Since the number of malaria cases is rising again, most of the increase occurring in the African region”. Do you mean “since then”?

Yes, it has been corrected.

3. Line 83: perhaps add that the recommended dose could be wrong -many antimalarials have been introduced at doses which were too low.

It has been added. The modified sentence reads now:

Low drug exposure can occur for various reasons including **suboptimal recommended dose from the registered product**, poor patient adherence, poor-quality medicines (either sub-standard or falsified medicines with reduced active ingredients), or inadequate absorption (e.g. acute vomiting shortly after drug administration) [15, 16].

4. Please define hyperparasitaemia (i.e. >100,000/uL) earlier in the paper.

The definition has been added at the first mention in the Methods section (page 7, line 150).

5. References 22-24 do support the case for the 100,000/uL “increased risk of treatment failure” threshold but it would be very helpful if there could either a figure or a description so that the reader could gauge the relationship between admission parasite density and risk of ssubsequent antimalarial treatment failure. I realise there are many caveats for each of the three referenced studies but, as this is such an important point, the parasitaemia -treatment failure risk does need to be presented in some way in this paper.

We have added a reference to White, 2009, and referred to Figure 6, when explaining underdosing in case of hyperparasitaemia (see response to Q6).

6. The introduction provides a strong case for the study. Perhaps add in a sentence to explain or differentiate the risk of underdosing from either low exposure (e.g. pregnancy) or because the standard duration of dosing is insufficient (e.g. hyperparasitaemia).

The following paragraph has been added:

**Antimalarial drug pharmacokinetics are usually altered during pregnancy, due to an expanded apparent volume of distribution, resulting in lower drug levels for any given dose. Another scenario resulting in underdosing is when the standard duration of dosing is not sufficient, which might be the case in treatment of uncomplicated Pf malaria patients presenting with hyperparasitaemia, usually defined as parasitaemia >100,000 per microlitre. The expected treatment effect is achieved with each dose, however due to the large parasite load and the first order parasite killing process, there are still parasite left in the body when the concentration of the drug drops below the minimum inhibitory concentration (see Figure 6 in [17]).**

7. Patients with parasite densities greater than 100,000/uL represent the largest proportion of potentially underdosed patients. The relationship between parasite density and therapeutic response at different levels of malaria transmission intensity and with different ACTs has not been well defined. Please discuss this. WWARN has by far the greatest data sets to identify where we need more information and how confident we can be about this threshold (or more correctly these thresholds). It seems to me that we need more trials specifically in hyperparasitaemic children to determine the threshold for prescribing a longer ACT treatment course?

We have expanded the relevant section in the discussion and now it reads as follows:

Although severely ill hyperparasitaemic patients are likely to be hospitalised and treated parentally, recognising a patient with isolated uncomplicated hyperparasitaemia is challenging as diagnosis is usually made by qualitative malaria RDTs and without microscopic confirmation of parasite density. These patients are thus likely to receive a standard oral ACT dosage regimen that may be insufficient to reduce their high parasite biomass thus increasing the risk of recrudescence [17]. **This problem could be rectified with use of semi-quantitative RDTs, which would allow to identify patients with parasitaemia above certain threshold. Although 100,000 parasites/microlitre have been used in the past to define hyperparasitaemia, the `optimal’ cutoffs may vary between different patient populations, different transmission intensity areas, and for different ACTs so further studies are needed to refine this definition**. Currently, even if the diagnosis of uncomplicated hyperparasitaemia is made, then the treatment remains problematic as evidence to date to support, e.g. increasing the ACT duration, is insufficient [14, 39].

8. Please discuss the difficulty in identifying “uncomplicated hyperparasitaemia” if current RDTs are used for diagnosis. Do we need semi-quantitative RDTs to rectify this problem?

This has been added to the discussion. Please see response to Q7.

Reviewer #2:

This is a paper describing estimates of malaria in risk groups that may have higher failure rates following ACT treatment and tries to estimate the number of treatment failures in these risk groups, suggesting that these may contribute to within host emergence of resistance. The assumptions of this analysis are not well supported and are so broad as to make the results somewhat unhelpful. There is no rigorous treatment of uncertainty.

We have restructured the paper, and have now moved most of the methods from the Supplementary Material to the Methods section and have expanded on the justifications of the assumptions made. We have also conducted further analyses, which, whenever possible, considered the uncertainty around the estimates of number of patients in different risk subpopulations and in the estimation of excess number of treatment failures.

Details are provided in the specific responses below.

9. Table 1 is presented as if there is no uncertainty.

We have now added twosupplementary tables (Supplementary Tables S11 and S12) to include the uncertainty limits around the point estimates of the number of malaria cases in subpopulations of interest (overall and per country), which were derived from the upper and lower estimates provided for each subpopulation within each country in the source databases (presented in Supplementary Table S9). The range values for the estimated proportion of children <5 years of age who were wasted were not available for five countries, and therefore for these countries only mean proportion was used in calculations. The mean hyperparasitaemia’s proportion was extracted from the published meta-analysis, and now we have added 95% CI for this proportion (as a marginal estimate adjusted for study-site estimated using a random effect logistic regression model) and included it in our calculations.

Similarly, calculations of the excess number of treatment failures presented in Table 3, were repeated for the most/least conservative scenario, using the lowest /highest number of patients within the subpopulations together with the corresponding lower /upper limit of the 95%CI for the risk of treatment failure. For the subgroups without risk estimates, the sensitivity analysis provides a measure of their impact on the overall estimates. These estimated ranges are now presented in the Supplementary Table S14.

Estimated ranges are included in the supplementary material, as it was impossible to add these numbers to the original tables and maintain nice formatting and readability. However, we now also refer to these supplementary tables in the results section, and quote uncertainty ranges in the text for the main results.

Details of calculations of limits of estimates are now presented in the methods section:

**Calculation of the number of patients within subgroups of interest was conducted using the reported point estimates, and using lower and upper limits (where available) to provide a measure of uncertainty in our estimates. For five countries, the range values were not available for the estimated proportion of children <5 years of age who were wasted, and therefore for these countries the reported point estimate was used in the calculation of uncertainty limits. Similarly, calculations of the excess number of treatment failures were repeated for the most/least conservative scenario, using the lowest /highest number of patients within the subpopulations together with the corresponding lower/upper limit of the 95%CI for the risk of treatment failure. For the subgroups without risk estimates, the sensitivity analysis provides a measure of their impact on the overall estimates. We refer to the lower and upper limits of our estimates as uncertainty range (UR).**

And have added the following limitations to the Discussion:

**We have attempted to provide a measure of uncertainty in the estimates of number of cases in population at risk of sub-optimal dosing and estimated the number of excess treatment failures, and have included upper and lower limits of the extracted parameter values in our calculations. However, this was not possible for all parameters. The ranges presented are therefore only an approximation of the extend of uncertainty in our predictions.**

10. In the real world, the estimates provided here are likely to be completely eclipsed by non-adherence to treatment and suboptimal dosing which is brushed aside with the statement: 100% coverage with ACTs and adherence with ACTs was assumed.

The objective of this study was to characterize the problem of suboptimal dosing in patients treated with WHO recommended ACTs at the manufacturer’s recommended dose. While we appreciate that patients who have not received adequate dosing due to no access to ACTs or due to non-adherence, are receiving suboptimal dosing, and are at the risk of increased treatment failure, this group of patients is not of interest of this study. We aim to evaluate the WHO-recommended ACTs regimen, rather than all sources of suboptimal dosing.

We have clarified our objective in the last sentence of Introduction, which now reads:

**This study aims to estimate the proportion of uncomplicated Pf malaria cases in endemic African countries treated with oral ACTs, as per current WHO guidelines, who are at risk of receiving sub-optimal dosing, and to estimate the fraction of treated patients likely to fail treatment because of sub-optimal dosing.**

We have also included the following paragraph in the Methods section:

**Since the objective of this study was to characterize the problem of suboptimal dosing in patients treated with oral ACTs as per current WHO guidelines, 100% adherence and 100% ACT coverage was assumed. This represents the best-case scenario, as patients with non-adherence or not receiving recommended ACTS are at risk of suboptimal doing, independently of their age or other risk factors.**

11. Here is a list of assumptions that are vague, uninformative or incorrect:

We have now moved the explanation of our assumptions from the supplementary material to the main text and have reworded them to clearly provide the justification. Our justification is provided below for each of the assumptions.

- Malaria risk was considered 4 times higher in the rural areas

This assumption was based on the published entomological inoculation rates (EIR) estimates for the sub-Saharan countries. For each of the three studies cited, we have calculated overall average EIR in urban and rural areas, and their ratio. The corresponding ratios were 6.25 (Robert, 2003), 3.03 (Hay 2005), 3.70 (Doumbe-Belisse, 2021), so we used ratio of 4 as a reasonable representation of the data. We have not estimated the confidence interval for this estimate.

- Proportion of risk groups within malaria patient populations was assumed to be the same as in the overall country population.

We agree with reviewer that this is a limitation of our work. However, we are not aware of any relevant data available in the literature. We believe however, that we have adjusted our calculations for the most important factors for which some data were available in literature.

Also, we would like to stress that all calculations in this analysis are meant to illustrate the extend of the risk of suboptimal dosing despite having received the recommended oral ACT treatment, and its possible consequences.

- Recrudescence in risk groups was based on unpublished reviews or assumed to be between 1.2-2.0
o Overweight – assumed to be 1.5

o Pregnant – assumed to be 1.5

We have not found any studies which have provided comparison of risk of treatment failure between pregnant or obese patients and patients without these risk factors. We have therefore decided to conduct a sensitivity analysis – and consider a range of possible values for HR corresponding to small (HR=1.2), medium (1.5) and high (HR=2.0) effect.

- Malaria cases are based on a model and are not validated

Number of malaria cases was calculated using the total number of reported cases from the 2021 WHO Malaria Report. The number of uncomplicated cases and their distribution across age groups was obtained using results from two modelling studies referred in the manuscript.

We appreciate that this approach has limitations. To our knowledge data on uncomplicated malaria cases or per age groups are not available per country, and this is the best available approach which allows us to perform country-specific estimation taking into account country characteristics.

This has been added to the study limitations section:

**Firstly, the number of uncomplicated malaria patients and their distribution across age structure was derived from modelling studies as only the estimated total number of malaria cases per country is available from WHO.**

- Hyperparasitemia is assumed to be a constant proportion of all cases

We have now included 95% confidence interval for the average proportion of patients with uncomplicated malaria who are hyperparasitaemic, derived from the cited meta-analysis, using random effects model adjusted for study-site.

- Only selected studies in Table S11 were used?

This is correct. We used estimates of risk of treatment failure for our subgroups of interest extracted from the systematic review of meta-analysis we have conducted in Epistemonikos. Those relevant (in grey in Table S13, previous Table S11) were used: one for children < 5 years old wasted compared to the same age group not wasted; and five comparing risk in patients with hyperparasitaemia >100000 parasites/µL to those without hyperparasitaemia. To clarify, this is now explained in the table heading.

We have now removed lines in Table S13 (previously Table S11) which referred to the increased risk in children <5 years of age without other risk factors, compared to other age groups, as this analysis on the advice from the reviewer is not presented anymore (see Q12).

- 100% coverage with ACTs and adherence with ACTs was assumed.

We have addressed this in response to Q10.

11. Note that the methods are two paragraphs, with a 30 page supplement that has to be scoured to really understand what they did.

We have now moved the description of methods and justification of our assumptions to the Methods section so we hope that the manuscript is more readable. We have also restructured Supplementary Material documents so it is easier accessible.

12. Suddenly at the end of the results we find this statement –
Three of the five IPD meta-analyses consistently identified that children <5 years of age with uncomplicated malaria but without the above-mentioned risk factors were also at the increased risk of treatment failure when compared to adults (HR 2.68 [95%CI: 1.87-3.85], supplementary material (p21-24) and were associated with an estimated additional number of failure rates of 1.050 - 4.202 million for ACPRs between 98% and 92%.
Which is not part of the methods or objectives, but with an estimated figure that is 2-10 times greater than any of the subgroups in Table 3.

We agree with the reviewer that this is confusing, and have removed this paragraph. We have also removed relevant lines in Table S13 (previously Table S11) which referred to the increased risk in children <5 years of age without other risk factors, compared to other age groups.

13. Taken together, these assumptions and methodological flaws render the results to be of limited value.

We hope we have provided enough details of the methodology so that the results would be considered valuable.
